# The Native Microbiome is Crucial for Offspring Generation and Fitness of *Aurelia aurita*

**DOI:** 10.1128/mBio.02336-20

**Published:** 2020-11-17

**Authors:** Nancy Weiland-Bräuer, Nicole Pinnow, Daniela Langfeldt, Anna Roik, Simon Güllert, Cynthia M. Chibani, Thorsten B. H. Reusch, Ruth A. Schmitz

**Affiliations:** a Molekulare Mikrobiologie, Institut für Allgemeine Mikrobiologie, Kiel University, Kiel, Germany; b Marine Ecology, GEOMAR Helmholtz Centre for Ocean Research Kiel, Kiel, Germany; University of Hawaii at Manoa

**Keywords:** *Aurelia aurita*, host, host fitness, microbiome, microbiota, reproduction

## Abstract

All multicellular organisms are associated with microbial communities, ultimately forming a metaorganism. Several studies conducted on well-established model organisms point to immunological, metabolic, and behavioral benefits of the associated microbiota for the host. Consequently, a microbiome can influence the physiology of a host; moreover, microbial community shifts can affect host health and fitness. The present study aimed to evaluate the significance and functional role of the native microbiota for life cycle transitions and fitness of the cnidarian moon jellyfish *Aurelia aurita*. A comprehensive host fitness experiment was conducted studying the polyp life stage and integrating 12 combinations of treatments with microbiota modification (sterile conditions, foreign food bacteria, and potential pathogens). Asexual reproduction, e.g., generation of daughter polyps, and the formation and release of ephyrae were highly affected in the absence of the native microbiota, ultimately resulting in a halt of strobilation and ephyra release. Assessment of further fitness traits showed that health, growth, and feeding rate were decreased in the absence and upon community changes of the native microbiota, e.g., when challenged with selected bacteria. Moreover, changes in microbial community patterns were detected by 16S rRNA amplicon sequencing during the course of the experiment. This demonstrated that six operational taxonomic units (OTUs) significantly correlated and explained up to 97% of fitness data variability, strongly supporting the association of impaired fitness with the absence/presence of specific bacteria. Conclusively, our study provides new insights into the importance and function of the microbiome for asexual reproduction, health, and fitness of the basal metazoan *A. aurita*.

## Abstract (precis)

All multicellular organisms are associated with a diverse and specific community of microorganisms; consequently, the microbiome is of fundamental importance for health and fitness of the multicellular host. However, studies on microbiome contribution to host fitness are in their infancy, in particular, for less well-established hosts such as the moon jellyfish *Aurelia aurita*. Here, we studied the impact of the native microbiome on the asexual reproduction and on further fitness traits (health, growth, and feeding) of the basal metazoan due to induced changes in its microbiome. We observed significant impact on all fitness traits analyzed, in particular, in the absence of the protective microbial shield and when challenged with marine potentially pathogenic bacterial isolates. Notable is the identified crucial importance of the native microbiome for the generation of offspring, consequently affecting life cycle decisions. Thus, we conclude that the microbiome is essential for the maintenance of a healthy metaorganism.

## INTRODUCTION

In recent years, a new conceptual framework—the metaorganism concept—was established, which is considering a metaorganism as the collective interactions among a multicellular host and its associated microbial species (1). The metaorganism concept focuses on the function and contribution (beneficial or detrimental) of the host-associated microbiota in a given environment, which depend on the identity, abundance, and activity of the microbes (2–4). The function of a specific microbe or microbial consortia is dynamic and depends on the developmental stage of the host, its age, reproductive state, or physiological condition (4–6). Several studies have recently found specific host-associated microbiota to contribute to host metabolism (7, 8), development (9), organ morphogenesis (10), pathogen protection and immunity (11, 12), behavior (13), environmental sensing and adaptation (5, 14–19), developmental transitions (20–23), and reproduction (24, 25). Conversely, in the absence of a proper microbial community, the functioning of the metaorganism may be compromised, which can result in various diseases; in humans, for instance, anxiety, depression, diabetes, cancer, obesity, and chronic inflammations are linked to microbiome imbalances (6, 26, 27).

Complex relationships and dependencies within a metaorganism are best investigated in controlled experiments that manipulate host microbiota composition (4), thereby enabling insights into the functional contributions of microbes to metaorganism function. The simplest and most drastic experimental setup is to compare hosts that are largely or completely lacking microbes (gnotobiotic or germfree, respectively) with those harboring their native microbiota (11, 28–31). Such studies on the well-documented Euprymna scolopes-*Vibrio* symbiosis demonstrated that only in the presence of their native microbiota (comprising the symbiont Vibrio fischeri), juvenile squid underwent a tissue remodeling of the external epithelial cell layer of the light organ (32, 33). In contrast, in squid raised in the absence of V. fischeri, tissue regression did not occur (34). Moreover, recolonization experiments using sterile animals in combination with available microbial isolates further enable assigning a certain function to specific microbes (11, 30, 35–37), determining colonization dynamics of microbes (30, 38, 39), and considering bacteria-bacteria interactions (11, 31, 40, 41). Until now, most studies on host-microbiota interactions were performed with well-established model organisms, such as the model plant Arabidopsis thaliana, the fruit fly Drosophila melanogaster, the nematode Caenorhabditis elegans, or mice (42, 43). However, their complexity and/or long generation times often limit studies on host-microbiota interactions (44). Novel model systems might lack some of the features making model organisms easy to investigate, such as low-cost and easy husbandry, short life cycles, high fertility, and genetic manipulability; however, they might enable to gain new insights into host-microbe interactions, as demonstrated by recent studies comparing the microbiota of a wide range of different metaorganisms (45, 46).

Here, we develop a member of the phylum Cnidaria as a new model system for metaorganism research among the basal metazoans. Our chosen model is the moon jellyfish *Aurelia aurita*, representing one of the most widely distributed Scyphozoa (47, 48). This species plays an influential role in the marine ecosystem, since they affect the structure of the planktonic food web and cause jellyfish blooms around the world due to their enormous stress tolerance, regeneration potential, and high reproduction output (49–53). Furthermore, *A. aurita* has a simple body plan with only two tissue layers, where host-microbe interactions can take place—the ectoderm and endoderm, separated by a jelly-like layer called the mesoglea. *A. aurita* possesses a complex life cycle. After sexual reproduction, *A. aurita* releases planula larvae, which settle on a suitable substratum and develop to the sessile, benthic polyp stage. *A. aurita* shows two forms of asexual reproduction—budding and strobilation. During budding, at one specific polyp site, a bud develops as an outgrowth due to repeated cell division to clonally form new individuals, which detach from the parent body when fully mature (54, 55). During strobilation, induced by environmental triggers, a transverse segmentation of the polyp body takes place. This mode of reproduction produces many offspring at a high rate that are key to understanding frequent jellyfish blooms (56). The process starts with preliminary morphological changes; in particular, the tentacles are reabsorbed. First transverse constrictions appear near the upper stalk; furthermore, the number of constriction sites increases and migrates down the body length, transforming the body into a sequence of segments. Those segments are released as ephyrae, the preform of juvenile medusa (52). The polyp foot remains adhered to the substrate and regenerates to a new polyp (52). In the present study, we were particularly interested in the role and impact of microbiota for regulating *A. aurita* complicated reproductive cycle involving very different life stages—mobile medusa, as a member of the marine plankton, and sessile polyp, as a member of the benthos (52, 57). Previously, we demonstrated specific bacterial community patterns for *A. aurita* life stages, which undergo significant restructuring during the polyp-jellyfish transition, strongly arguing for an important functional role of the associated microbiota, particularly in the context of the life cycle (29). A crucial microbial impact on animal development has already been demonstrated for the bacterial induction of settlement and metamorphosis of many marine invertebrate larvae (58), such as sponges (59), cnidarians (60), ascidians (61), and bryozoans (62). The settlement of planula larvae of the scyphozoans *A. aurita* (60) and Cassiopea andromeda (63) or the hydrozoan Hydractinia echinata (20) is prevented in the absence of microbes; in contrast, the presence of bacteria induces the settlement and development of each of those animals (64). A comprehensive host fitness experiment was designed and conducted with the benthic polyp life stage of *A. aurita* to evaluate the importance and function of the microbiome on fitness traits such as asexual reproduction and survival. The bacterial communities of polyps were manipulated in 12 different treatments that included polyps with native microbiota or sterile ones kept in artificial seawater (sterile or with native microbiota), feeding with Artemia salina (unmanipulated or sterile), and experimental infection with potentially pathogenic bacteria correlating with the concomitant changes in microbial community composition. Our overall aim was to assess the importance of the native microbiome for health and, particularly, for the generation of offspring, ultimately proposing that the microbiome is essential for the maintenance of a healthy metaorganism.

## RESULTS

**FIG 1:**
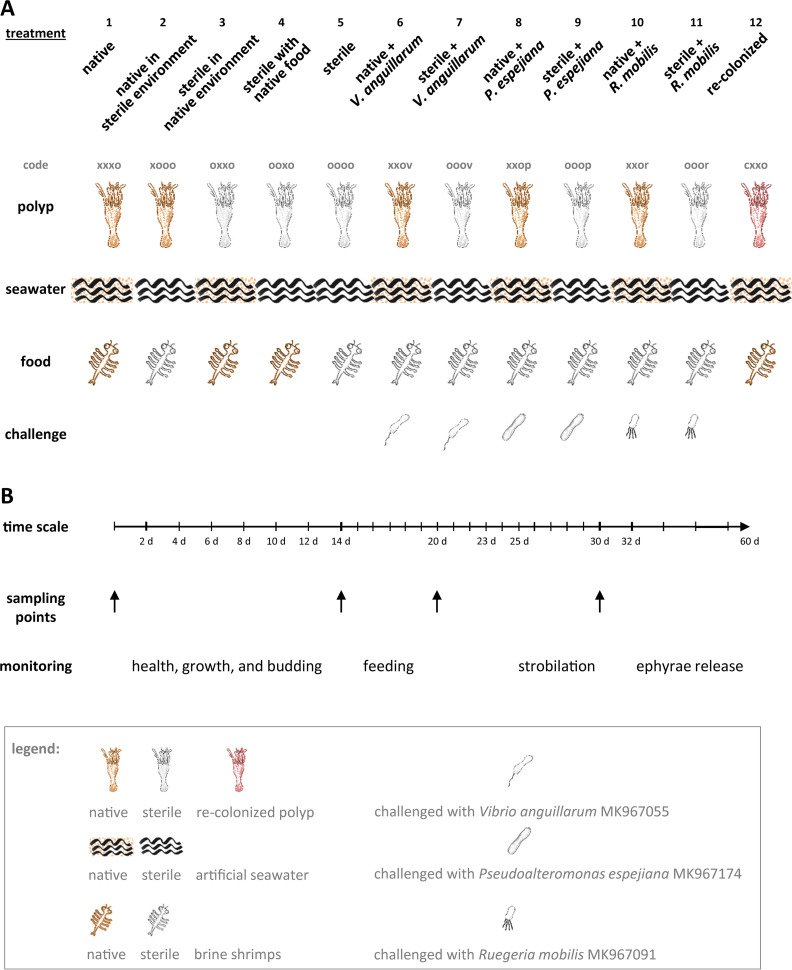
Study design for host fitness experiment. (A) Each treatment comprises a certain combination of polyp, seawater, and food regarding their microbial composition. Selected potential pathogenic bacteria were used for the microbial challenge. A code is introduced for each treatment describing the presence “x” or absence “o” of microbes in the order polyp, ambient water, and food; microbial challenge is labeled with “v” for Vibrio anguillarum, “p” for Pseudoalteromonas espejiana, and “r” for Ruegeria mobilis; recolonized polyps are labeled with” c.” (B) An experimental schedule comprising the time scale of the experiment with monitoring events and sampling points for subsequent 16S rRNA amplicon sequencing is illustrated.

A comprehensive host fitness experiment with *A. aurita* polyps with high numbers of replicates (96) for each treatment was conducted in 48-well plates, where polyps were kept in 1 ml artificial seawater, to evaluate the importance and function of the microbiome on host fitness traits, i.e., asexual reproduction, survival, growth, and feeding (the experimental design and abbreviations are summarized in Fig. 1). To examine the general effects of microbiota, native polyps that harbor a native diverse microbial community (polyps were kept under artificial lab conditions) were compared with sterile polyps generated by using an antibiotic mixture and recolonized polyps. As major treatment factors, native as well as sterile polyps were kept in artificial seawater (sterile or with native microbiota), fed with *Artemia salina* (sterile or unmanipulated), and challenged with potentially pathogenic bacteria previously isolated from *A. aurita* polyps and ambient seawater (Vibrio anguillarum, Pseudoalteromonas espejiana, and Ruegeria mobilis) to evaluate the impact of microbiota on the resistance of the metaorganism against bacterial infections (Fig. 1).

### Influence of the microbiota on asexual reproduction of *A. aurita*.

**FIG 2:**
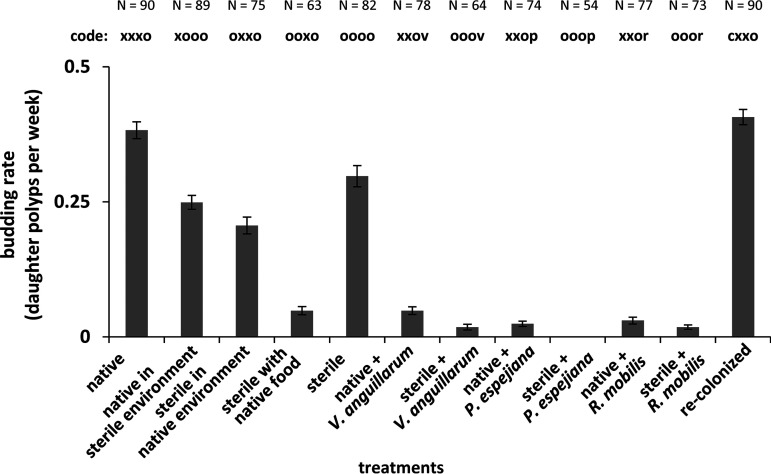
Asexual reproduction of *A. aurita* polyps as the daughter polyp generation. Budding was followed every 48 h for 14 days by monitoring the generation of daughter polyps of healthy and harmed polyps. Budding rate was calculated as daughter polyp generation per week.

The generation of daughter polyps was monitored every 48 h over 14 days. Native as well as recolonized polyps showed a similar budding rate of 0.4 daughter polyps per week (0.38 ± 0.02 and 0.41 ± 0.01, respectively), whereas sterile polyps generated 0.3 ± 0.02 daughter polyps/week, representing a statistically significant decrease of 21% (*P* value of 0.0003) (Fig. 2; see also Fig. S1 and Table S1C in the supplemental material). Environmentally challenged polyps (treatments 3 and 4) had further decreases in budding rates, with decreases for sterile polyps in native environment by 45% and sterile polyps incubated with unmanipulated food by 87% (Fig. 2 and S1; Table S1D) (treatment 3: *t* = 4.0, *P* value < 0.0002; treatment 4: *t* = 6.0, *P* value < 0.0001). Budding rates also decreased remarkably when polyps were infected with potential pathogens, resulting in almost completely impaired generation of daughter polyps, in particular, when challenged with *P. espejiana* (Fig. 2 and S1; Table S1E) (F = 71.3, *P* value for permutational multivariate analysis of variance [*P*_PERMANOVA_] < 0.0001).

10.1128/mBio.02336-20.1 FIG S1 Daughter polyp generation of *A. aurita* polyps. Budding was followed every 48 h for 14 days by monitoring the generation of daughter polyps of healthy and harmed polyps. Numbers of generated daughter polyps are shown as percentages after 14 days. Download FIG S1, DOCX file, 0.1 MB. Copyright © 2020 Weiland-Bräuer et al. 2020 Weiland-Bräuer et al. This content is distributed under the terms of the Creative Commons Attribution 4.0 International license.

10.1128/mBio.02336-20.6 TABLE S1 PERMANOVA tests. (A) Overview of PERMANOVA study design. (B) All variables were tested under three test designs, A, B, and C. (C) PERMANOVA test A with full data set (4-factorial, partly nested). (D) PERMANOVA test B with polyp-seawater-food data set (3-factorial, partly nested). (E) PERMANOVA test C with bacterial challenge data set (2-factorial, fully crossed). ns, not significant. Download Table S1, DOCX file, 0.1 MB. Copyright © 2020 Weiland-Bräuer et al. 2020 Weiland-Bräuer et al. This content is distributed under the terms of the Creative Commons Attribution 4.0 International license.

**FIG 3:**
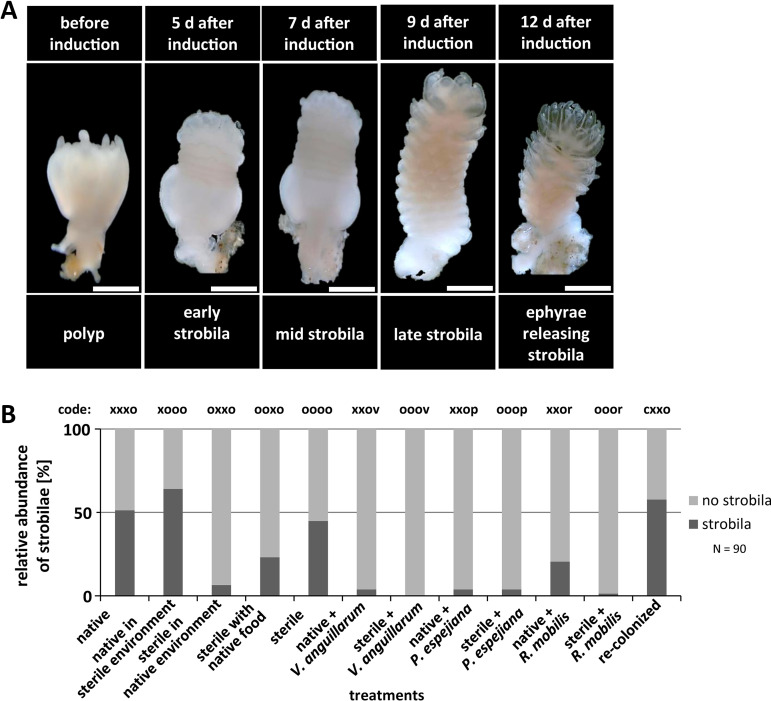
Asexual reproduction of *A. aurita* polyps as the generation of strobilae. Strobilation of polyps was induced using 5 μM 5-methoxy-2-methyl indole. (A) Original photographs show progress of segmentation for polyps under native conditions. Scale bars correspond to 1 mm. (B) Segmentation of polyps was monitored each day, and the numbers of generated strobilae were determined.

**FIG 4:**
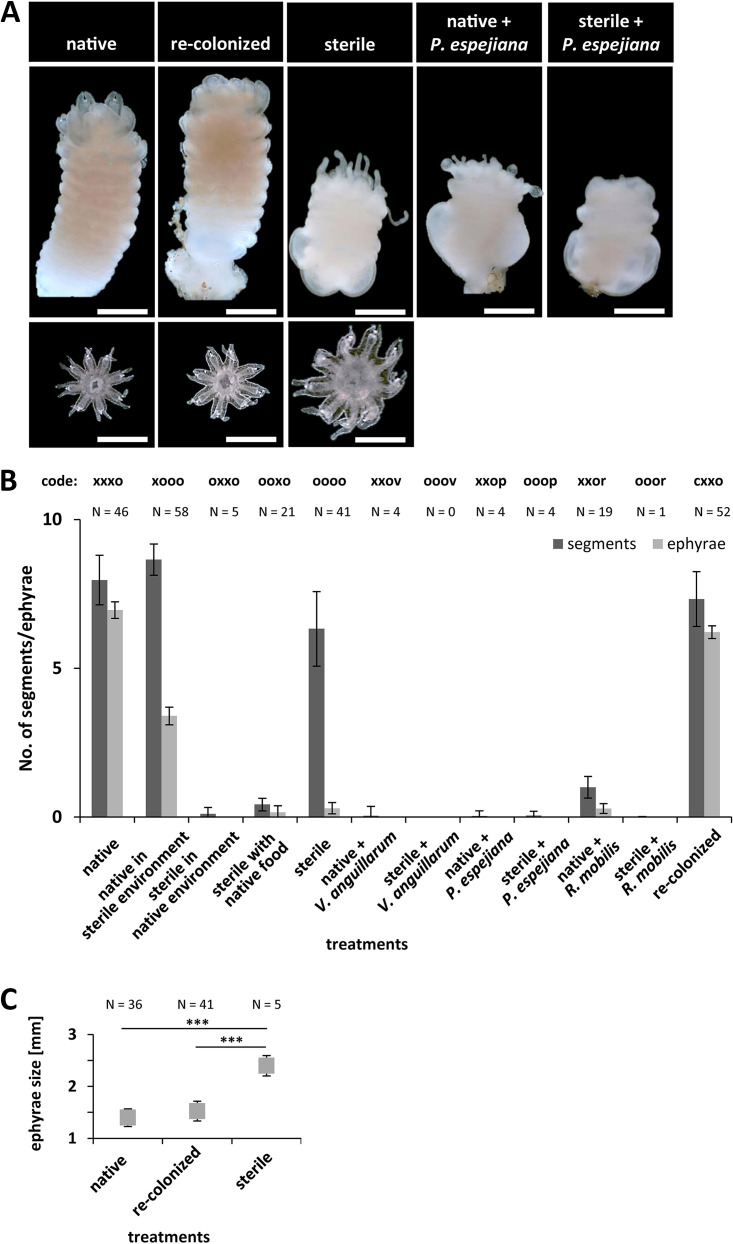
Segmentation of polyps and ephyra release. Strobilation of polyps was induced using 5 μM 5-methoxy-2-methyl indole. (A) Original photographs show phenotypical appearance and segmentation of strobilae under selected representative treatments. Photographs of released ephyrae are shown at the bottom at 15 days postinduction. Scale bars correspond to 1 mm. (B) Numbers of segments per strobila and numbers of released ephyrae were monitored each day for a maximum of 4 weeks. (C) Diameters of released ephyrae under native, sterile, and recolonized conditions were quantified. ***, *P* < 0.001.

In a parallel experimental setup, strobilation of polyps was induced by adding the synthetic strobilation inducer 5 μM 5-methoxy-2-methyl indole (Fig. 1) (58). Strobilation induction was monitored each day, and strobila phenotypes as well as number of segments were monitored and detected beginning on day 5, when native polyps initiated segmentation (early strobila) (Fig. 3A; Table S2). Native polyps showed full segmentation and shaping as late strobila on day 9 (Fig. 3A; Table S2). Specifically, 51% of native and 58% of recolonized polyps showed initiation of segmentation on day 5 after induction (Fig. 3B; Table S2). Even 64% of native polyps in the sterile environment formed strobilae, which showed typical segmentation (Fig. 3B). However, the formation of strobilae was delayed, with early strobilae appearing after 8 days and late strobilae after 17 days. In contrast, 45% of sterile polyps showed segmentation (Fig. 3B; Table S1C) (*t* = 10.7, *P* value < 0.0001) (Table S2), but remarkably, phenotypically abnormal strobilae were formed (Fig. 4A). Phenotypical abnormalities were manifested by colorless, minimized, and thickened strobilae without absorbed tentacles (see examples in Fig. 4A). Polyps, which were environmentally challenged (treatments 3 and 4) showed massively reduced formation of strobilae, and the few ones observed were phenotypically abnormal (colorless, minimized, and thickened strobilae with inflated calyx) (Fig. 4). Likewise, infected polyps (treatments 6 to 11) showed almost no formation of strobilae (Fig. 3B; Table S1E) (F = 121.7, *P*_PERMANOVA_ < 0.0001), resulting in almost completely halted segmentation (Fig. 4A). Furthermore, subsequent ephyra release was detected for all treatments, beginning on day 12 after induction of strobilation, when native strobilae began to release ephyrae (Fig. 3A and 4A; Table S2). Native and recolonized strobilae formed approximately eight segments and finally released seven ephyrae with a size of 1.5 ± 0.1 mm in diameter after 12 to 14 days after induction (Fig. 4B; Table S2). Native polyps in a sterile environment formed nine segments but deferred release of only three ephyrae (Fig. 4B; Table S2). Sterile polyps were only slightly affected in induced segmentation (45% strobilae with a mean of six segments); however, an abnormal strobila phenotype was continuously detected, resulting in massively impaired ephyra release (Fig. 4; Table S1C) (F = 171.9, *P*_PERMANOVA_ < 0.0001). Moreover, only 15% of the formed sterile strobilae were able to release only a single ephyra of increased size (2.4 ± 0.2 mm, diameter increase of 70%) (Fig. 4C). Ultimately, infected polyps showed almost no segmentation; consequently, no ephyrae were released (Fig. 4; Table S1C) (F = 10.2, *P*_PERMANOVA_ < 0.0001). These findings strongly argue that strobilation and consequently ephyra release are massively interrupted in the absence of the native polyp microbiota as well as when infected with potential pathogens, ultimately resulting in halted generation of offspring.

10.1128/mBio.02336-20.7 TABLE S2 Summary of mean fitness status of *A. aurita* polyps and strobilae for all treatments. Heat map summarizes the significant decreases measured within the treatment groups compared to that in the native treatment. Based on individual results after host fitness experiments, monitored fitness parameter survival, growth, budding, feeding, strobilation, and ephyra release are categorized as unaffected (orange), slightly affected (purple), and crucially affected (red) for each treatment. Additionally, timelines for generation of strobilae and ephyra release are depicted as days of first appearance after strobilation induction with the synthetic inducer. na, not applicable. Download Table S2, DOCX file, 0.1 MB. Copyright © 2020 Weiland-Bräuer et al. 2020 Weiland-Bräuer et al. This content is distributed under the terms of the Creative Commons Attribution 4.0 International license.

### Microbial impact on the fitness traits survival, growth, and feeding.

**FIG 5:**
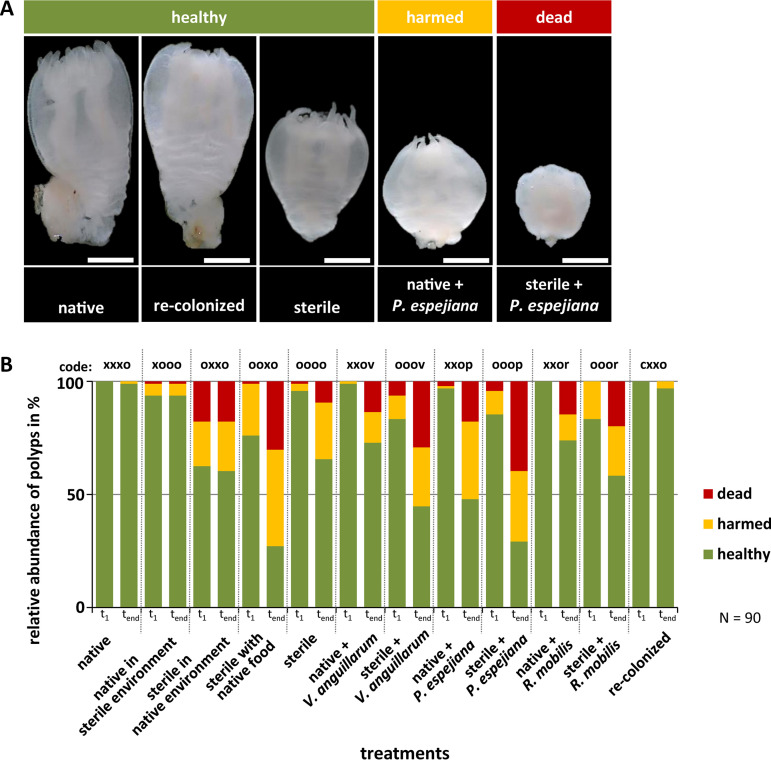
Impact of microbes on health of *A. aurita* polyps. (A) Original photographs show examples of healthy, harmed, and dead polyps after 14 days for representative selected treatments. Scale bars correspond to 0.5 mm. (B) Percentages of healthy, harmed, and dead polyps monitored based on phenotypical appearance of polyps and presence of tentacles are shown for all treatments monitored after 48 h (*t*_1_) and 14 days (*t*_end_) comprising 90 biological replicates each.

In parallel with asexual reproduction (budding), further important fitness-correlated traits, in particular, survival and growth of *A. aurita* polyps, were monitored every 48 h over 14 days. Based on the overall phenotypical appearance of polyps and their tentacles, three health conditions were differentiated, namely, “healthy” (normal polyp phenotype, contracting tentacles), “harmed” (polyp phenotype impaired, tentacles degenerated), and “dead” (degenerated or no polyp) (see “Host fitness experiment” for detailed definition on phenotype characteristics), which are exemplified in Fig. 5A. Recolonized polyps showed similar survival rates as native polyps without an impaired phenotype (99%, corresponding to 89 ± 1 polyps, and 97%, corresponding to 87 ± 3 polyps, respectively), whereas 37% of sterile polyps were affected in health after 14 days (*t*_end_) (Fig. 5B; Table S1C) (*t* = 3.8, *P* value < 0.0001). Native polyps were able to maintain their associated native microbiota (see below and Fig. 5) when kept in a sterile environment at least over the short period during the experiment (code xooo, only 5% polyps were affected) (Fig. 5B). However, sterile polyps were affected in survival or at least showed malformation when kept under native environmental conditions (39% affected) (Table S1D) (*t* = 2.8, *P* value < 0.0008) and were affected even more when fed with *A. salina* harboring their native microbiota (78% affected) (Fig. 5B; Table S1D) (*t* = 5.6, *P* value < 0.0001). Environmentally challenged polyps (treatments 3 and 4) (Fig. 1) showed significantly increased malformation and mortality already at the onset of the experiment, which worsened over time. Remarkable health loss, with up to 76% impaired polyps, was also detected when challenging with potentially pathogenic bacteria which were previously isolated from *A. aurita* and ambient water (65) (Fig. 5B; Table S1C) (*P* value < 0.0001). The survival rate of polyps was significantly affected by each bacterial challenge (Table S1E) (F = 15.9, *P*_PERMANOVA_ < 0.0001), with the exception of *R. mobilis* infection (Table S1E) (pairwise tests, *P* value was not significant). In more detail, we observed that unmanipulated native polyps were affected by infection with potentially pathogenic bacterial isolates during the course of the experiment, whereas sterile polyps were affected early on. Furthermore, *R. mobilis* and V. anguillarum (belonging to *A. aurita* native microbiota) affected the health/survival of native polyps only slightly (28%, of which 16% were dead, and 29%, of which 14% were dead, respectively) (Table S1E) (*P* value < 0.0001) compared to the effect of *P. espejiana* (56%, of which 19% were dead) (Table S1E) (*P* value < 0.0001) (Fig. 5B). A significant interaction of the type of ambient water (unmanipulated or sterile) as well as *Artemia* supplied as food (unmanipulated or sterile) was further identified (Table S1E) (F = 7.6, *P*_PERMANOVA_ < 0.0005 and F = 34.5, *P*_PERMANOVA_ < 0.0001, respectively), indicating that specific bacteria differed in their challenge depending on both water and food quality.

**FIG 6:**
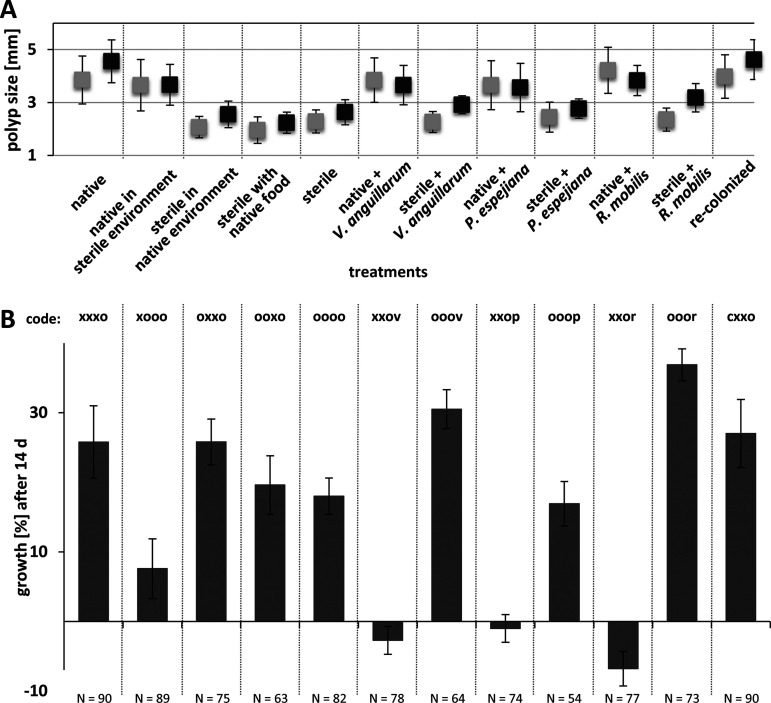
Growth rate of *A. aurita* polyps. Growth of polyps was followed every 48 h for 14 days by measuring the polyp size (length plus width) of healthy and harmed polyps. (A) Mean polyp start sizes (▪) and end sizes after 14 days (▪) per treatment. (B) Growth rates were calculated as percentages after 14 days.

**FIG 7:**
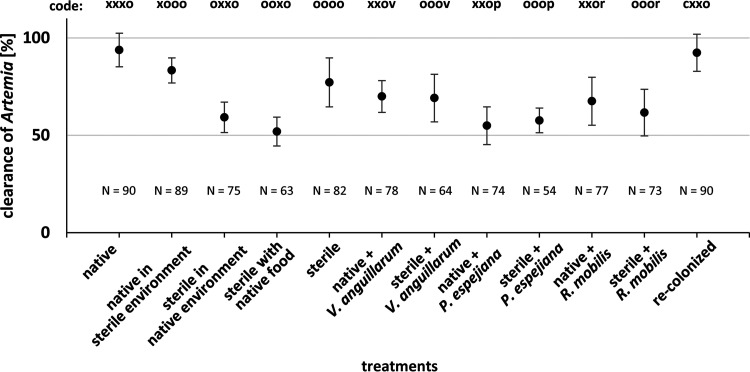
Clearance of *Artemia salina* by *A. aurita* polyps. Feeding rate as clearance of *A. salina* in percentages. Single polyps were incubated for 1 h with 20 *A. salina*. After incubation, remaining *A. salina* were counted to calculate the feeding rate of healthy and harmed polyps for all treatments. Feeding rates are displayed as means of all monitoring days (Σ5).

Healthy and harmed polyps were also analyzed with respect to growth and feeding rates. Native polyps had a mean polyp size (length plus width) of 3.85 ± 0.91 mm, whereas sterile polyps showed reduced start sizes of 2.3 ± 0.43 mm (Fig. 6). Native and recolonized polyps showed similar initial sizes and further similar growth rates (Fig. 6). Analyses indicated that growth was primarily affected in sterile artificial seawater and when infected with Vibrio anguillarum and Ruegeria mobilis (Fig. 6; Table S1). Feeding rates of healthy and harmed polyps were assessed in week three of the experiment on the five following days (Fig. 7 and S2). Native polyps had a mean clearance rate of 93.8% ± 8.6%, thus, similar to recolonized polyps (92.4% ± 9.5%), whereas sterile polyps showed a 77.2% ± 12.5% clearance rate (Fig. 7 and S2; Table S1C) (*t* = 12.5, *P* value <0.0001). Feeding rates were significantly reduced when treatments affected the overall health of polyps (Fig. S2). Analyses further disclosed a significant effect on the presence of the associated polyp microbiota as well as an effect of bacterial infection on feeding rates (Fig. 7 and S2). Native polyps were further affected in effective feeding when kept in sterile artificial seawater (Table S1) (*P* values < 0.0001).

10.1128/mBio.02336-20.2 FIG S2 Clearance of *Artemia salina* by *A. aurita* polyps. Feeding rate as clearance of *A. salina* in percentages. Single polyps were incubated for 1 h with 20 *A. salina*. After incubation, remaining *A. salina* were counted to calculate the feeding rate of healthy as well as harmed polyps for all treatments (corresponding codes in parentheses). Compared are the results from day 1 (I_1_) and day 5 (*t*_end_). Download FIG S2, DOCX file, 0.2 MB. Copyright © 2020 Weiland-Bräuer et al. 2020 Weiland-Bräuer et al. This content is distributed under the terms of the Creative Commons Attribution 4.0 International license.

### Microbial community shifts correlate with impaired fitness traits.

**FIG 8:**
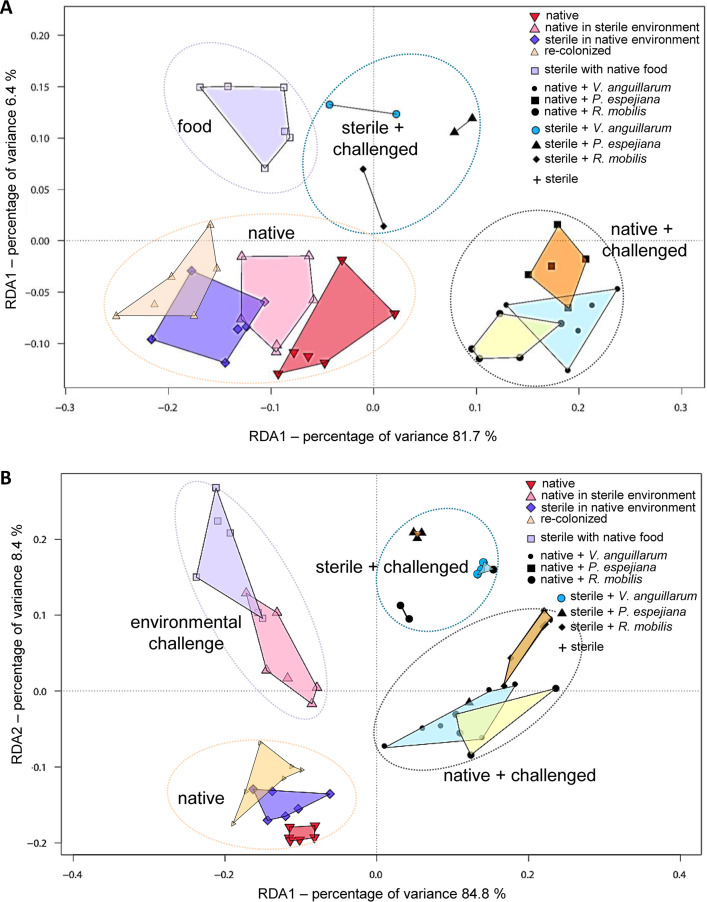
Microbial composition of *A. aurita* polyps and strobilae under selected microbial challenges. Composition of microbiota associated with *A. aurita* polyps after 14 days of microbial challenge (12 different treatments) during the host fitness experiment. Microbial communities were analyzed by sequencing the amplified V1-V2 region of 16S bacterial rRNA genes. Redundancy analysis plots of Hellinger-transformed OTU abundances. Each point represents the whole microbial community of the respective sample; replicates of one sample group are framed by polygons. Microbial community patterns of polyps (A) and strobilae (B) are shown.

**FIG 9:**
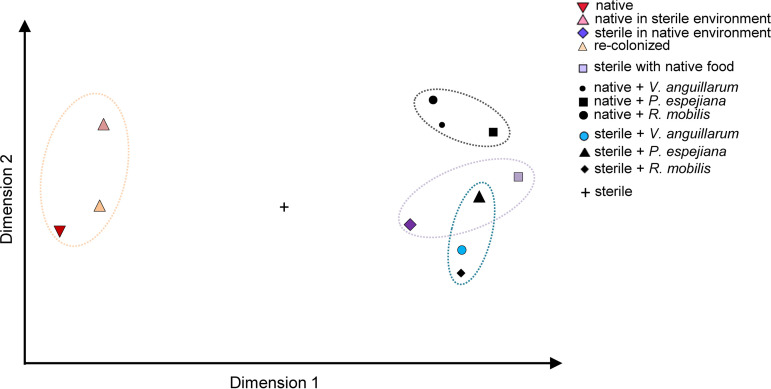
Multivariate ordination results (nonmetric multidimensional scaling [NMDS] based on Bray-Curtis) of average fitness traits.

To correlate the impaired asexual reproduction, survival rates, growth, and feeding with changes in the microbial community of the native *A. aurita* microbiota, 16S V1-V2 amplicon sequencing was performed during the course of the experiment (see Table S3). Antibiotic-treated polyps and strobilae, developed from sterile polyps, were indeed shown to be sterile (Fig. 8 and S3, S4, and S5). In line with our previous report (29), the present analysis further showed restructuring of the microbial community during polyp-jellyfish transition (Fig. S3, S4, and S5). Native and recolonized polyps showed similar bacterial community patterns due to successful reconstitution (Fig. 8A and S4; Table S3). However, relative abundances of community members were different, resulting also in identification of specific indicator operational taxonomic units (iOTUs) explaining the microbial communities (Fig. S4; Table 1). Moreover, changes in the native microbial community composition induced by the experimental treatments were identified (Fig. 8 and S3). Notable are the higher abundances of unclassified (uncl.) *Gammaproteobacteria*, uncl. *Cyanobacteria*, and *Melitea* as well as the lower abundance of *Arcobacter* in the native microbiota of the animals (Fig. S3). Redundancy analysis revealed four distinct microbial community clusters resulting from the respective manipulation (native, environmental challenged, native plus challenged, and sterile plus challenged) for polyps as well as strobilae (Fig. 8). Indicator OTU analyses further revealed bacterial taxa, which significantly correlated either positively or negatively with fitness traits (Tables 1 and 2). For instance, the presence of OTU0142 (uncl. *Prevotella*) was linked to essentially impaired fitness traits, whereas OTU0015 (uncl. *Neptuniibacter*) and OTU0003 (uncl*. Alteromonas*) might be beneficial bacteria (Tables S4 and S5). Moreover, OTU0057, OTU0178, and OTU0336 (*Olleya*, *Rothia*, and *Gemella*, respectively) were exclusively beneficial for strobilation or, in contrast, the presence of OTU0110 and OTU0144 (*Corynebacterium* and *Balneola*, respectively) resulted in halted transverse constriction (Table S5). Finally, the multivariate ordination analysis visually summarizes the observed fitness effects (Fig. 9), which mirror the identified microbial community clusters from redundancy analysis (RDA) (Fig. 8), ultimately correlating the observed fitness effects with microbial community shifts.

**TABLE 1:** Indicator operational taxonomic units

Indicator OTU^*a*^	Taxonomic classification^*b*^	*P* value
Polyps		
Native		
OTU0016	Melitea salexigens	0.001
OTU0083	Uncl. *Glaciecola*	0.041
OTU0120	Melitea salexigens	0.005
OTU0220	Uncl. *Sinobacteraceae*	0.007
Recolonized		
OTU0015	Neptuniibacter caesariensis	0.003
OTU0121	Uncl. *Oceanospirillaceae*	0.001
OTU0130	Uncl. *Bacteriovorax*	0.001
OTU0131	Uncl. *Flavobacteriales*	0.001
OTU0134	Uncl. *Pseudoalteromonas*	0.001
OTU0179	Uncl. *Ruegeria*	0.001
Native microbiota		
OTU0009	Uncl. *Cyanobacteria*	0.004
OTU0073	Uncl. *Gammaproteobacteria*	0.001
OTU0126	Uncl. *Gammaproteobacteria*	0.001
Native in sterile environment		
OTU0078	Uncl. *Vibrio*	0.009
OTU0267	Uncl. *Gammaproteobacteria*	0.020
Sterile in native environment		
OTU0054	Lewinella cohaerens	0.006
OTU0082	Uncl. *Flavobacteriaceae*	0.001
OTU0128	Uncl. *Polaribacter*	0.003
OTU0141	Uncl. *Balneola*	0.005
OTU0218	Uncl. *Fluviicola*	0.001
OTU0241	Uncl. *Polaribacter*	0.004
Sterile with native food		
OTU0048	Uncl. *Plesiocystis*	0.008
OTU0065	Uncl. *Arcobacter*	0.002
OTU0097	Uncl. *Saprospiraceae*	0.001
OTU0099	Uncl. *Oleibacter*	0.005
OTU0116	Uncl. *Oleibacter*	0.005
OTU0138	Uncl. *Gammaproteobacteria*	0.007
OTU0145	Uncl. *Lactococcus*	0.005
OTU0163	Microbacterium aurum	0.011
Sterile		
Not detected		
Native plus V. anguillarum		
OTU0107	Uncl. *Chromobacterium*	0.001
OTU0155	Uncl. *Flavobacteriaceae*	0.009
OTU0258	Uncl. *Proteobacteria*	0.040
OTU0355	Uncl. *Proteobacteria*	0.016
OTU0203	Uncl. *Vibrio*	0.001
Sterile plus V. anguillarum		
OTU0203	Uncl. *Vibrio*	0.003
Native plus *P. espejiana*		
OTU0021	Uncl. *Bizionia*	0.002
OTU0052	Uncl. *Proteobacteria*	0.001
OTU0166	Micrococcus luteus	0.001
OTU0175	Rothia mucilaginosa	0.003
OTU0200	Uncl. *Bacteria*	0.001
OTU0098	Uncl. *Pseudoalteromonas*	0.004
Sterile plus *P. espejiana*		
OTU0098	Uncl. *Pseudoalteromonas*	0.004
Native plus *R. mobilis*		
OTU0077	Uncl. *Nannocystis*	0.005
OTU0093	Uncl. *Cytophagales*	0.003
OTU0142	Uncl. *Prevotella*	0.001
OTU0169	Uncl. *Mogibacteriaceae*	0.015
OTU0179	Uncl. *Ruegeria*	0.002
Sterile plus *R. mobilis*		
OTU0179	Uncl. *Ruegeria*	0.002
Strobilae		
Native		
OTU0029	Uncl. *Shewanella*	0.007
OTU0142	Uncl. *Prevotella*	0.006
OTU0169	Uncl. *Mogibacteriaceae*	0.002
Recolonized		
OTU0108	Uncl. *Gammaproteobacteria*	0.019
OTU0167	Uncl. *Gammaproteobacteria*	0.002
OTU0192	Uncl. HTTC	0.005
Native in sterile environment		
OTU0162	Uncl. *Staphylococcus*	0.034
OTU0260	Uncl. *Neptuniibacter*	0.005
OTU0268	Uncl. *Lewinella*	0.001
Sterile in native environment		
OTU0051	Uncl. *Proteobacteria*	0.033
OTU0052	Uncl. *Proteobacteria*	0.032
Sterile with native food		
OTU0094	Uncl. *Alteromonas*	0.016
OTU0099	Uncl. *Oleibacter*	0.026
OTU0343	Uncl. *Oceanospirillaceae*	0.003
Sterile		
Not detected		
Native plus V. anguillarum		
OTU0138	Uncl. *Gammaproteobacteria*	0.043
OTU0163	Uncl. *Microbacterium*	0.018
OTU0203	Uncl. *Vibrio*	0.048
OTU0252	Uncl. *Alteromonas*	0.005
OTU0324	Uncl. *Arcobacter*	0.009
Sterile plus V. anguillarum		
OTU0203	Uncl. *Vibrio*	0.004
Native plus *P. espejiana*		
OTU0098	Uncl. *Pseudoalteromonas*	0.003
OTU0173	Uncl. *Haliangiaceae* (*Kofleriaceae*)	0.017
Sterile plus *P. espejiana*		
OTU0098	Uncl. *Pseudoalteromonas*	0.004
Native plus *R. mobilis*		
OTU0077	Uncl. *Nannocystis*	0.013
OTU0082	Uncl. *Flavobacteriaceae*	0.002
OTU0103	Uncl. *Streptococcus*	0.005
OTU0175	Uncl. *Rothia*	0.009
OTU0179	Uncl. *Ruegeria*	0.006
Sterile plus *R. mobilis*		
OTU0179	Uncl. *Ruegeria*	0.022

a Indicator OTUs identified as key operational taxonomic units for the respective treatments.

b Uncl., unclassified.

**TABLE 2:** Correlation of operational taxonomic units with fitness data^*a*^

OTU	Taxonomic classification	Adj. *R*^2^	SS(trace)	Pseudo-F	*P* value	Prop	Cumul	Res df
OTU0142	Uncl. *Prevotella*	0.55	41.51	13.11	**0.00**	0.59	0.59	9
OTU0167	Uncl. *Gammaproteobacteria*	0.65	8.83	3.59	**0.01**	0.13	0.72	8
OTU0015	Neptuniibacter caesariensis	0.76	7.99	4.79	**0.01**	0.11	0.83	7
OTU0106	Uncl. *Corynebacterium*	0.84	5.00	4.49	**0.01**	0.07	0.90	6
OTU0065	Uncl. *Arcobacter*	0.90	3.10	4.34	**0.01**	0.04	0.95	5
OTU0267	Uncl. *Gammaproteobacteria*	0.93	1.70	3.64	**0.03**	0.02	0.97	4
OTU0003	Uncl. *Alteromonas*	0.95	0.91	2.87	0.08	0.01	0.99	3
OTU0110	Uncl. *Gammaproteobacteria*	0.97	0.58	3.04	0.12	0.01	0.99	2
OTU0085	SC3-56	0.99	0.29	3.23	0.25	0.00	1.00	1
OTU0355	Uncl. *Proteobacteria*	0.99	0.00	0.00	1.00	0.00	1.00	1

a Distance based linear model (DistLM) analysis and sequential tests show predictors (operational taxonomic units [OTUs]) explaining the host fitness data structure in the best model (adjusted *R*^2^ = 0.99, *R*^2^ = 1.00, RSS [residual sums of squares] = 0.09, number of fitted variables = 10 OTUs) with a significance level of 0.05, which are marked in bold. Uncl., unclassified; Adj. *R*^2^, adjusted *R*^2^; SS(trace), sum of squares; Pseudo-F, multivariate analogue to Fisher’s F test; Prop, proportion of variance explained; Res df, residual degrees.

10.1128/mBio.02336-20.3 FIG S3 Microbial community composition of *A. aurita* in the experimental treatment groups. Microbial communities were analyzed by sequencing the V1-V2 region of 16S bacterial rRNA genes. OTU abundances were summarized at the genus level and normalized by the total number of reads per sample. Bar plots are grouped according to sample type, each group including 2 to 6 replicates. Microbial community patterns of polyps (A) and strobilae (B) are shown. Download FIG S3, DOCX file, 0.6 MB. Copyright © 2020 Weiland-Bräuer et al. 2020 Weiland-Bräuer et al. This content is distributed under the terms of the Creative Commons Attribution 4.0 International license.

10.1128/mBio.02336-20.4 FIG S4 Microbial composition of recolonized polyps. Compositions of microbiota associated with native and recolonized *A. aurita* polyps as well as of the generated native microbiota for recolonization of sterile polyps. Microbial communities were analyzed by sequencing the V1-V2 region of 16S bacterial rRNA genes. (A) OTU abundances were summarized at the genus level and normalized by the total number of reads per sample. Bar plots are grouped according to sample type, each group including 6 replicates. (B) Redundancy analysis plots of Hellinger-transformed OTU abundances. Each point represents the whole microbial community of the respective sample, replicates of one sample group are framed by polygons. (C) Results of pairwise tests in beta diversity analysis. Tests were conducted for specified comparisons. df, degree of freedom; v, variance. Download FIG S4, DOCX file, 0.1 MB. Copyright © 2020 Weiland-Bräuer et al. 2020 Weiland-Bräuer et al. This content is distributed under the terms of the Creative Commons Attribution 4.0 International license.

10.1128/mBio.02336-20.8 TABLE S3 Results of pairwise tests in beta diversity analysis. Permutation test for RDA under reduced model was conducted with permutations (number of permutations, 9,999) for all comparisons, but only specified comparisons are shown. Model: RDA (formula = OTUhel ∼ group, data = metaDataSub); df = 0.321, v = 6.663, *P* value = 0.00001. Download Table S3, DOCX file, 0.1 MB. Copyright © 2020 Weiland-Bräuer et al. 2020 Weiland-Bräuer et al. This content is distributed under the terms of the Creative Commons Attribution 4.0 International license.

10.1128/mBio.02336-20.9 TABLE S4 Correlation of operational taxonomic units (OTUs) with the fitness status of *A. aurita* polyps. OTUs listed with their taxonomic classification are correlated with the fitness status of unaffected/healthy (orange), slightly affected/marginally harmed (purple), and crucially affected/essentially harmed (red) of *A. aurita* polyps. At least 50% of the biological replicates analyzed were associated with the respective OTU. Download Table S4, DOCX file, 0.1 MB. Copyright © 2020 Weiland-Bräuer et al. 2020 Weiland-Bräuer et al. This content is distributed under the terms of the Creative Commons Attribution 4.0 International license.

10.1128/mBio.02336-20.10 TABLE S5 Correlation of operational taxonomic units (OTUs) with the segmentation of *A. aurita* polyps and ephyra release. Unaffected (orange), slightly affected (purple), and crucially affected (red) formation of strobilae and further ephyra release are correlated with respective listed OTUs. At least 50% of the biological replicates analyzed were associated with the respective OTU. Download Table S5, DOCX file, 0.1 MB. Copyright © 2020 Weiland-Bräuer et al. 2020 Weiland-Bräuer et al. This content is distributed under the terms of the Creative Commons Attribution 4.0 International license.

## DISCUSSION

The findings of the present study clearly demonstrate that the native microbiota is particularly crucial for asexual reproduction of *A. aurita* as well as for survival, growth, and feeding (Fig. 1 and 7; see also Table S2 in the supplemental material). While the absence of the microbiota clearly impairs *A. aurita* fitness traits, they can be restored when sterile polyps are recolonized with the native microbiota, strongly arguing for a significant bacterial impact on host fitness. Although we cannot completely exclude off-target impacts of antibiotics on cell division or metabolism, recolonized polyps showed a normal phenotype despite antibiotic treatment; further indications are obtained from transcriptomic data (unpublished data). Moreover, the associated native microbiota of native polyps appears to be stably maintained even in a sterile environment. The decrease of growth rates in the absence of the microbiota and when additionally infected with potentially pathogenic bacteria confirms our assumption of the importance of a balanced microbiota (Fig. 6). However, the finding that growth rates did not decrease or even increase when bacteria were present in the environment (unmanipulated environment and infection with bacteria, treatments 3, 7, 9, and 11) compared to rates for polyps in a sterile environment (treatment 2) indicates that bacteria from the environment most likely act as an additional food source for the polyps (Fig. 6) (29, 66, 67). Sterile polyps in a completely sterile environment already showed reduced fitness traits, but infection with foreign (treatment 4) or potentially pathogenic bacteria (treatments 6 to 11) caused a strong inhibition of asexual reproduction as well as an increase in mortality/malformation (Fig. 9; Table S2). The results of the present study demonstrate that the native microbiota is crucially required for asexual reproduction of the moon jellyfish by budding. Budding rates were correlated with the observed health status of polyps and thus diminished in absence and after manipulation of the native microbiota (Fig. 2). Likewise, bacteria have been found to have profound effects on budding of the fresh water polyp Hydra viridissima (68). Sterile animals were also unable to produce buds and multiply asexually, which is the exclusive type of asexual reproduction for *Hydra*, because they lack the process of strobilation (69). Generation of daughter polyps was rescued by inoculation with native bacterial associates of *Hydra* (68). Furthermore, our study revealed that the native microbiota is fundamental for correct transverse constriction of *A. aurita* during the process of strobilation, which ultimately results in the release of ephyrae as offspring (Fig. 4). Generation and release of offspring are halted in the absence of the associated microbiota and when infected with potential pathogens. The requirement of an associated microbiota for reproduction was recently also demonstrated for the fruit fly *Drosophila* (70). It was shown that the fly gut stimulated bacterial diversity, which, in turn, enhanced development, aging, and reproduction of the host. At the current stage, we can only speculate on the bacterial contribution to *A. aurita* reproduction at the molecular level, but a bacterial metabolite or small molecule appears likely to be fundamental. As hypothesized, the native microbiota most likely acts as a protective shield and supports defense against facing potentially pathogenic bacteria, since polyp fitness was impaired in the absence of the microbiome and was even more crucial when additionally infected with potentially pathogenic bacteria. Multivariate ordination analysis (Fig. 9) of observed fitness effects perfectly matches with microbial community patterns identified in RDAs (Fig. 2). Indicator OTU analysis points to key bacteria, which are correlated with health or impaired fitness of *A. aurita* (Tables 1, S4, and S5).

The role of microbes in pathogen defense is exemplified for various invertebrates. In corals, commensal bacteria defend the attachment of a pathogen on the mucus and protect against the bleaching-causative Vibrio shiloi (71, 72). Moreover, the endosymbiont *Spiroplasma* protects *Drosophila* against nematodes by producing RIP toxins (73). The ability of associated microbes to interfere with pathogen colonization and growth is known as colonization resistance (74–76) and has also been documented across vertebrates and plants (77, 78). While the concept of colonization resistance is not new, its underlying molecular mechanisms, ranging from direct microbe-microbe competition (e.g., niche competition or direct antagonism) to indirect induction or priming of host metabolism or immunity, are less understood (79). Nevertheless, one prominent example from vertebrates clearly demonstrates the role of bacteria in combating infectious diseases. Nowadays, patients suffering from severe diarrhea caused by Clostridium difficile infection can be successfully treated using fecal transplants from healthy donors, which harbor beneficial microbes required for gut homeostasis (80).

In conclusion, our study contributes to the growing body of evidence that associated microbes are an essential part of the host phenotype, influencing fitness, and are thus ecologically important traits of their hosts (81–84). The functional contributions of native microbiomes to host fitness are manifold, comprising, for instance, pathogen protection, nutrient allocation, development, behavior, adaptation, detoxification, and mating selection (reviewed in reference 85). The observed decline in reproductive output and fitness of the jellyfish *A. aurita* upon disturbance of its natural microbiota in the present study clearly points to the importance of an organism-specific associated microbiota, ultimately facilitating a healthy metaorganism. Our results further emphasize the importance of using novel model organisms to study host-microbe interactions and highlight the importance of the microbiome for health and fitness of a host in general. We further assume that bacterial diversity and species abundances within the microbiome are balanced on the polyp, most likely due to interbacterial (11, 40, 41, 83) and host-microbe interactions (31, 86) as well as host genotype (87, 88). However, the molecular mechanism of the microbial contribution to *A. aurita* fitness, in particular, to asexual reproduction, has to be elucidated in the future. Impending identification of the underlying mechanism and involved molecules also has a potential for application. Concisely, asexual reproduction ensures enormous multiplication of polyps in an environment by budding or massive production of juvenile medusae via strobilation (56). This fast and frequent multiplication can lead to jellyfish blooms, which significantly impact ecological community composition and structure by altering carbon, nitrogen, and phosphorus cycling and reducing available prey for higher predators (89, 90). In the future, the knowledge of microbial molecules triggering massive offspring generation might lead to the development of compounds interfering with the asexual reproduction of *A. aurita*, which will ultimately be useful to control jellyfish blooms.

## MATERIALS AND METHODS

### *Aurelia aurita* polyp husbandry and generation of sterile polyps.

Husbandry and generation of sterile polyps are described in detail by Weiland-Bräuer et al. (29). Briefly, polyps of subpopulation North Atlantic (Roscoff, France) were kept more than 10 years in the lab in 2-liter plastic tanks in 3% artificial seawater (ASW) (tropical sea salts; Tropic Marin). Polyps were fed twice a week with freshly hatched *Artemia salina* (HOBBY, Grafschaft-Gelsdorf, Germany). Generation of sterile polyps was performed with antibiotic mixture added to sterile (filtered through 0.22-μm Durapore membrane filters; Merck Millipore, Darmstadt) artificial seawater (Provasoli’s antibiotic mixture with final concentrations of 360,000 U/liter penicillin G, 1.5 mg/liter chloramphenicol, 1.8 mg/liter neomycin, and 9,000 U/liter polymyxin B; all components from Carl Roth, Karlsruhe, Germany). Generation of sterile *Artemia salina* was similarly conducted. Eggs were hatched in sterile ASW with Provasoli’s antibiotic mixture (complemented with 3.5 mg/liter nystatin and 3.5 mg/liter amphotericin B). The absence of bacteria was confirmed by plating homogenized animals on marine bouillon agar plates (Carl Roth). After incubation at 19°C for 5 days, the CFU were determined. Absence of CFU indicated successful antibiotic treatment. Additionally, the full-length bacterial 16S rRNA gene was amplified using GoTaq polymerase (Promega, Madison, WI, USA) and primer set 27F (5′-AGAGTTTGATCCTGGCTCAG-3′)/1492R (5′-GGTTACCTTGTTACGACTT-3′) with genomic DNA isolated from putative sterile polyps and *A. salina* using a Wizard genomic DNA purification kit according to the manufacturer’s instructions (Promega). Samples without successful amplification were graded as sterile.

### Bacterial growth conditions.

Bacteria (Vibrio anguillarum, Pseudoalteromonas espejiana, and Ruegeria mobilis; GenBank accession numbers MK967055, MK967174, and MK967091, respectively) for microbial challenge during host fitness experiments were isolated from *A. aurita* polyps of subpopulations from the Baltic Sea and North Atlantic as well as 3% ASW (30 practical salinity units [PSU]), respectively, as described in reference 65. Strains were grown in marine bouillon (MB; Carl Roth, Karlsruhe, Germany) at 30°C and 120 rpm to a turbidity optical density at 600 nm (OD_600_) of 0.8. Bacterial cell numbers were detected using a Neubauer count chamber (91), and 10^5^ cells/ml were complemented with 5% dimethyl sulfoxide (DMSO) (1-ml aliquots) to store at −80°C before using 10 μl of 10^5^ cells/ml in 1 ml ASW per well during host fitness experiments. Concisely, Vibrio anguillarum isolated from *A. aurita* Baltic Sea polyps is widely distributed in marine and estuarine environments around the world (92–94). It is a global causative agent of vibriosis in marine fish and shellfish species, because it can efficiently grow and proliferate under environmental stress (95). Pseudoalteromonas espejiana isolated from artificial seawater is a ubiquitous bacterium abundantly found in marine environments and often associated with marine multicellular organisms (96). *P. espejiana* is able to degrade polymers and induces metamorphosis of Hydractinia echinata hydroid larvae (20, 97). Ruegeria mobilis was exclusively isolated from the polyp life stage (65). *R. mobilis* has primarily been isolated from marine aquaculture, where the tropodithietic acid (TDA)-producing strains have probiotic potential due to the inhibition of fish pathogens (98). V. anguillarum and *R. mobilis* belong to *A. aurita*’s native microbiota but are known as a potential pathogen and beneficial bacterium, respectively. *P. espejiana* is a nonnative bacterium.

### Recolonization of sterile polyps.

Sterile polyps were used for recolonization with generated native *A. aurita* polyp microbiota. For generation of native microbiota, native polyps were transferred from 2-liter husbandry tanks into 1.5 ml reaction tubes (polyps were not fed for at least 3 days before transposition) and washed three times with 1 ml sterile ASW. Polyps (10 per reaction tube) were homogenized with a motorized pestle (KONTES; DWK Life Sciences, Wertheim, Germany) and resuspended in 1 ml sterile ASW. The homogenate was subsequently filtered through 3.1-μm filters (Lab Logistic Group, Meckenheim, Germany) to remove eukaryotic cells. Prior to conducting the host fitness experiment, 10 μl of the filtrate (approximately 2.5 × 10^7^ cells/ml) was added to a single polyp in 1 ml ASW (48-well plate) and incubated for 48 h at 19°C. Polyps were washed three times with 1 ml sterile ASW to remove noncolonized bacteria. Additionally, prepared native microbiota (filtrate) and recolonized polyps (after 48 h of incubation) were used for bacterial 16S rRNA amplicon sequencing.

### Host fitness experiment.

Single native and sterile polyps of the subpopulation from the North Atlantic were transferred from 2-liter husbandry tanks to 48-well plates with 1 ml 3% ASW per well. Polyps settled to the bottom of the wells prior to the experiment start. For each treatment, 96 replicates were used. Twelve different treatments comprising combinations of polyp, food, and ambient seawater in terms of microbial composition (native, polyps maintained in ASW in the lab; sterile, antibiotic [AB] treated, challenged) were conducted to elucidate the impact of microbes on the fitness of the host (Fig. 1). Briefly, polyps either harbored their native microbiota (lab conditions) or were made sterile using an antibiotic mixture (see above). Moreover, one of the 12 treatments comprised sterile polyps, which were recolonized with generated native *A. aurita* polyp microbiota. Native or sterile artificial seawater (30 PSU, filtered through 0.22-μm filters) was used as ambient water, and *Artemia salina* with its native microbiota or sterile brine shrimps were applied as food. Native as well as sterile polyps were additionally infected with selected bacteria Vibrio anguillarum, Pseudoalteromonas espejiana, and Ruegeria mobilis. Six different fitness traits (survival, growth, feeding, budding, strobilation, and ephyra release) were analyzed using the stereomicroscope Novex Binokulares RZB-PL Zoom-Mikroskop 65.500 (Novex, Arnhem, Netherlands) with a high-definition multimedia interface (HDMI)/HD camera as follows. The survival/malformation of polyps was assessed every 48 h for 14 days based on overall phenotypical appearance of polyps and presence of tentacles and categorized as healthy, harmed, and dead (see Fig. 5). Healthy polyps showed typical morphology comprising an attached stalk and a stretched calyx with fully developed and extended tentacles. Polyps were rated as harmed when they lost at least two of those criteria, mostly showing a shrunken calyx and contracted or even absorbed tentacles. Dead polyps were of a shrunken roundish morphology or even dispersed. The health of polyps was compared between the first monitoring after 48 h (*t*_1_) and at the end of experiment after 14 days (*t*_end_). The growth of polyps was documented every 48 h for 14 days by measuring the length and width of the polyps, which were still alive (healthy and harmed). Mean start (*t*_1_) sizes (length plus width) and end sizes after 14 days (*t*_end_) of polyps were compared per treatment, and growth rates were calculated in percentages after 14 days for the remaining replicates. Moreover, budding as one form of asexual reproduction of *A. aurita* polyps, where daughter polyps are generated, was monitored for 14 days (in parallel to health and growth). Numbers of daughter polyps were counted for all alive (healthy and harmed) polyps, and the corresponding budding rate was calculated as daughter polyp generation per week. In addition, on days 15 to 19, feeding of alive polyps (healthy and harmed) was monitored each day. Therefore, freshly hatched *Artemia salina* were harvested from 500-ml brood containers in a harvesting container with an *Artemia* sieve (0.15-mm mesh). *A. salina* were washed three times with sterile ASW on the mesh and concentrated to 50 ml. A single polyp was fed with 20 *A. salina* (in 50 μl of sterile ASW) and incubated for 1 h before counting remaining *A. salina.* A clearance rate of *A. salina* was calculated per treatment in percentages for each day (day 1 [*t*_1_] up to day 5 [*t*_end_]). Finally, a new set of polyps (96 replicates per treatment) was prepared as mentioned above in 48-well plates, with a single polyp in 1 ml ASW corresponding to treatments shown in Fig. 1. Strobilation of polyps was induced by adding 5 μM 5-methoxy-2-methyl indole to the ambient water on the three following days (involving daily washing). Afterwards, polyps were washed on day 4 with the respective ambient water. Strobilation induction was monitored each day, and strobila phenotypes as well as number of segments were detected beginning on day 5, when native polyps began segmentation (early strobila). Native polyps showed full segmentation and shaping as late strobila on day 9. Recently, 5-methoxy-2-methyl indole was demonstrated to act as the temperature-dependent synthetic activator of strobilation in *A. aurita* (58). The selected procedure of chemical induction enables the reduction of the complexity of the strobilation process to a minimum, particularly, for investigating the impact of the microbiome and not the environmental triggers during reproduction. This procedure is more time efficient than the natural induction by lowering the temperature (approximately 1 month at 8°C). Ephyra release was monitored each day after first appearance, and numbers of released ephyrae were detected beginning on day 12, when native strobilae began to release ephyrae. Ephyra release was monitored for the next 4 weeks.

In addition to the monitoring of fitness traits, 16S rRNA amplicon sequencing was performed to analyze potential changes in the microbial community composition based on the treatments, ultimately correlating observed fitness traits with changes in microbial community patterns. Therefore, six polyps were removed from the 48-well plates before the experimental start (*t*_0_) for DNA isolation and subsequent 16S rRNA amplicon sequencing (90 replicates remaining for each treatment at *t*_0_). After 14 days (*t*_end_) of experiment, further six polyps were removed for 16S rRNA analysis. Furthermore, six polyps were also removed from the second set of polyps before induction of strobilation (*t*_0_). Likewise, six late strobila (removed on day 10 after strobilation induction) per treatment were removed for sequence analysis.

### Data analysis of host fitness parameters.

Factor effects were assessed for each fitness variable (i.e., counts of harmed and dead polyps, growth rate in percent, clearance rate in percent, budding rate in percent, strobilae count, and ephyra production in percent) using univariate permutational analysis of variance (99). All variables were tested under three test designs as outlined in Table S1. PERMANOVAs were performed on Euclidian resemblance matrices calculated from log_2_ (x + 1)-transformed data (100) and were based on 9,999 permutations of residuals under a reduced model and type III partial sums of squares using Primer-E V6. (100). Within each significant factor, pairwise *post hoc* tests followed, providing insight into differences between the treatment groups (Table S1).

### Nucleic acid isolation.

Eukaryotic as well as bacterial DNA from single *A. aurita* polyps was isolated using a Wizard genomic DNA purification kit (Promega, Madison, WI, USA) according to the manufacturer’s protocol.

### Illumina sequencing.

Bacterial DNA was isolated as described above and used for the generation of PCR amplicon libraries using uniquely barcoded primers flanking the V1-V2 hypervariable regions (27F–338R; V1_A_Pyro_27F [5′-CGTATCGCCTCCCTCGCGCCATCAGTCAGAGTTTGATCCTGGCTCAG-3′] and V2_B_Pyro_27F [5′-CTATGCGCCTTGCCAGCCCGCTCAGTCAGAGTTTGATCCTGGCTCAG-3′]) with fused MiSeq adapters and heterogeneity spacers in a 20-μl PCR using Phusion high-fidelity DNA polymerase (New England Biolabs) (101). Reactions included the following components: 4 μl of HF buffer, 0.4 μl deoxynucleoside triphosphates (dNTPs; 200 μM each), 0.8 μl each of forward and reverse primers (2 μM), 0.2 μl Phusion Hot Start II high-fidelity DNA polymerase, and 2 μl DNA (20 ng). PCRs were conducted with the following cycling conditions (98°C, 30 s; 30 × [98°C, 9 s; 55°C, 60 s; 72°C, 90 s]; 72°C, 10 min; 10°C, infinity). Amplicons were size checked and purified using a MinElute gel extraction kit (Qiagen). Purified amplicons were quantified using a Quant-iT PicoGreen kit (Invitrogen), and equal amounts of the purified PCR products were pooled for subsequent Illumina sequencing as indicated by band intensity and measured with Quant-iT PicoGreen kit. Amplicon sequencing was performed on the Illumina MiSeq platform with v3 chemistry (2 × 300 cycle kit) according to the manufacturer’s instructions at Max-Planck Institute for Evolutionary Biology in cooperation with S. Künzel.

### 16S rRNA data processing and bioinformatics.

All steps of sequence processing were conducted with the program mothur v1.39.5 according to the MiSeq standard operating procedure (SOP). In brief, 16S rRNA amplicon reads were aligned against the bacterial SILVA database, which was previously trimmed to the 16S V1-V2 rRNA gene regions to improve accuracy of the alignment (align.seqs). Subsequently, a random subset of 1,000 sequences per sample was generated to eliminate bias due to unequal sampling effort (sub.sample). The aligned and subsampled sequences were then classified using the Greengenes database v13_8_99 (classify.seqs) and reads assigned to the lineages *Archaea*, Chloroplast, Mitochondria, and unknown, were removed. The remaining sequences were used to compute a distance matrix (dist.seqs) for binning sequences into operational taxonomic units (OTUs) by average neighbor clustering (cluster.split). Five hundred eleven OTUs at a 97% similarity threshold (roughly corresponding to species level) distinction were considered. All downstream computations were performed in R v2.15.1 (102) as described in reference 29. Indicator OTU analysis was also performed in R. Mothur-shared OTU tables were imported into R using the phyloseq package (103). Subsequently, the multipatt function of the indicspecies package (104) was used to identify OTUs that were significantly associated with the replicate groups. For this analysis, only the original replicate groups, as provided in the group file, were considered, and 500 permutations were conducted. Significantly associated OTUs were summarized for each group, and their taxonomic classification was assigned. Furthermore, relationships of bacterial OTU abundance with host fitness variables in the treatment groups were evaluated using distance based linear models (DistLM) as implemented in Primer-E V6. Prior to analysis, host fitness data were averaged across the treatment groups, normalized, and aligned with square root-transformed OTU abundance data (100). The DistLM routine was performed using Euclidian distances and implementing a forward selection procedure with 999 permutations and adjusted *R*^2^ criterion. Additionally, Spearman rank correlation coefficients were obtained for the predictors.

### Data availability.

Sequence data were deposited under the NCBI BioProject PRJNA633008 comprising locus tag prefixes SAMN14930335 to SAMN14930451.

10.1128/mBio.02336-20.5 FIG S5 Microbial composition of strobilae. Compositions of microbiota associated with native *A. aurita* polyps before strobilation induction and native late strobilae 9 days after induction of strobilation with 5 μM 5-methoxy-2-methyl indole. Microbial communities were analyzed by sequencing the V1-V2 region of 16S bacterial rRNA genes. (A) OTU abundances were summarized at the genus level. (B) Redundancy analysis plots of Hellinger-transformed OTU abundances (df = 0.136, v = 9.652, *P* value = 0.005). Download FIG S5, DOCX file, 0.2 MB. Copyright © 2020 Weiland-Bräuer et al. 2020 Weiland-Bräuer et al. This content is distributed under the terms of the Creative Commons Attribution 4.0 International license.
