# Supplementary material for: The Native Microbiome is Crucial for Offspring Generation and Fitness of Aurelia aurita
Source: mBio. 2020 Nov 17;11(6):e02336-20. doi: 10.1128/mBio.02336-20 (PMC7683396; doi:10.1128/mBio.02336-20)
Supplement: FIG S1 [file mBio.02336-20-sf001.docx]

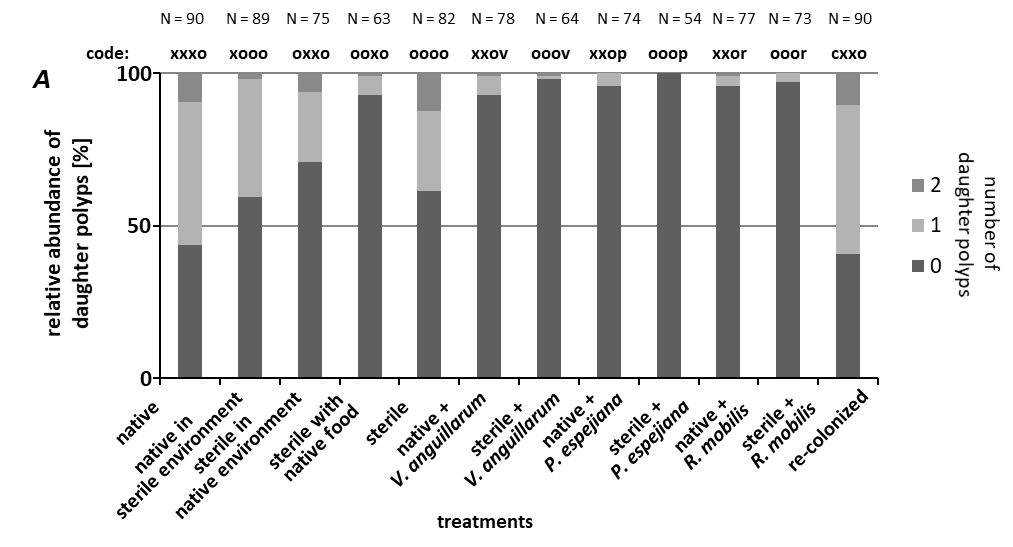


**Fig. S1: Daughter polyp generation of *A. aurita* polyps.** Budding was followed every 48 h for 14 d by monitoring the generation of daughter polyps of healthy and harmed polyps. Number of generated daughter polyps are shown in percentages after 14 d.
