# Supplementary material for: The Native Microbiome is Crucial for Offspring Generation and Fitness of Aurelia aurita
Source: mBio. 2020 Nov 17;11(6):e02336-20. doi: 10.1128/mBio.02336-20 (PMC7683396; doi:10.1128/mBio.02336-20)
Supplement: TABLE S1 [file mBio.02336-20-st001.docx]

**Tab. S1: PERMANOVA tests.** (***A***) Overview of PERMANOVA study design, (***B***) all variables were tested under three test designs A, B, C. (***C***) PERMANOVA test A with full data set (4-factorial, partly nested). ns, not significant (***D***) PERMANOVA test B with polyp-seawater-food data set (3-factorial, partly nested). ns, not significant (***E***) PERMANOVA test C with bacterial challenge data set (2-factorial, fully crossed). ns, not significant

***A***

| Cross tabulation of full study design | | | | | |
| --- | --- | --- | --- | --- | --- |
| Treatment group (code) | Polyp  (S, sterile) | Seawater (S, sterile) | Food  (S, sterile) | Bacterial challenge (Pe, *Pseudomonas espejiana*; Va, *Vibrio anguillarum*; Rm, *Ruegeria mobilis*) | |
| 1 (xxxo) | *native* | *native* | *native* | *none* | |
| 3 (oxxo) | *sterile* | *native* | *native* | *none* | |
| 4 (ooxo) | *sterile* | *sterile* | *native* | *none* | |
| 8 (xxop) | native | native | sterile | Pe | |
| 10 (xxor) | native | native | sterile | Rm | |
| 6 (xxov) | native | native | sterile | Va | |
| 2 (xooo) | *native* | *sterile* | *sterile* | *none* | |
| 9 (ooop) | sterile | sterile | sterile | Pe | |
| 11 (ooor) | sterile | sterile | sterile | Rm | |
| 7 (ooov) | sterile | sterile | sterile | Va | |
| 5 (oooo) | *sterile* | *sterile* | *sterile* | *none* | |
| PERMANOVA-A: All data = 4 factors; NOTE: Bacterial Challenge is nested in Food (sterile) | | | | | |
| PERMANOVA-B: *Polyp-Seawater-Food data, i.e., 3-factorial: Food is nested in Seawater* | | | | | |
| PERMANOVA-C: Bacterial Challenge data, i.e., 2-factorial: fixed in a fully crossed design | | | | | |

***B***

|  | PERMANOVA-A | *PERMANOVA-B* | PERMANOVA-C |
| --- | --- | --- | --- |
| Data Subset | treatment groups 1 – 11  (all data) | treatment groups  1, 2, 3, 4, 5 | treatment groups  2, 5, 6 ,7, 8, 9, 10, 11 |
| Factors | Polyp*Seawater*Food \| BacChallenge(Seawater*Food) | Polyp*Seawater*Food Food(Seawater) | Polyp*BacChallenge |
| Interaction | Polyp*BacChallenge(Seawater*Food) | Polyp*Seawater | Polyp*BacChallenge |
| Design | Bacterial Challenge is nested in Food*Seawater | Food is nested in Seawater | Factors fixed and fully crossed |

***C***

|  | Factors | | | | | | | | Interactions | | |
| --- | --- | --- | --- | --- | --- | --- | --- | --- | --- | --- | --- |
| **Fitness variable** | **Polyp** | | **Seawater** | | **Food** | | **Bacterial Challenge (SexAr)** | | **PoxCh(SexAr)** | | |
|  | **Pseudo-F** | **P(perm)** | **Pseudo-F** | **P(perm)** | **Pseudo-F** | **P(perm)** | **Pseudo-F** | **P(perm)** |  | **Pseudo-F** | **P(perm)** |
| **Survival**  **(Harmed and Dead Polyp Count)** | 14,259 | **0,0001** | no test | no test | no test | no test | 16,095 | **0,0001** | Interaction * | 3,8889 | **0,0006** |
| **Growth** | 3,0598 | 0,0769 | no test | no test | no test | no test | - | ns | Interaction * | 3,0006 | **0,0306** |
| **Feeding** | 155,53 | **0,0001** | no test | no test | no test | no test | 54,556 | **0,0001** | Interaction * | ns | ns |
| **Budding** | 13,55 | **0,0001** | no test | no test | no test | no test | 56,082 | **0,0001** | Interaction * | 2,4572 | 0,0616 |
| **Strobila production** | 114,78 | **0,0001** | no test | no test | no test | no test | 90,102 | **0,0001** | Interaction * | 14,111 | **0,0001** |
| **Ephyrae release** | 171,98 | **0,0001** | no test | no test | no test | no test | 10,2 | **0,0001** | Interaction * | 7,129 | **0,0001** |

| **Fitness variable** | **Effect** | **Comparison** | **Global** | |
| --- | --- | --- | --- | --- |
|  |  |  | **t** | **P(perm)** |
| **Survival (Harmed and Dead Pol Count)** | **Polyp** | **native polyp, sterile polyp** | 3,7761 | **0,0001** |
| **Survival (Harmed and Dead Pol Count)** | **Bacterial Challenge (SexAr)** | **none, Va** | 4,8357 | **0,0001** |
| **Survival (Harmed and Dead Pol Count)** | **Bacterial Challenge (SexAr)** | **none, Pe** | 7,5224 | **0,0001** |
| **Survival (Harmed and Dead Pol Count)** | **Bacterial Challenge (SexAr)** | **none, Rm** | 5,0663 | **0,0001** |
| **Survival (Harmed and Dead Pol Count)** | **Bacterial Challenge (SexAr)** | **Va, Pe** | 1,4854 | 0,1083 |
| **Survival (Harmed and Dead Pol Count)** | **Bacterial Challenge (SexAr)** | **Va, Rm** | 1,5846 | 0,0795 |
| **Survival (Harmed and Dead Pol Count)** | **Bacterial Challenge (SexAr)** | **Pe, Rm** | 2,6819 | **0,0016** |
| **Growth** | **Polyp** | **native polyp, sterile polyp** | 1,7492 | 0,0819 |
| **Feeding** | **Polyp** | **native polyp, sterile polyp** | 12,471 | **0,0001** |
| **Feeding** | **Bacterial Challenge (SexAr)** | **none, Va** | 5,4774 | **0,0001** |
| **Feeding** | **Bacterial Challenge (SexAr)** | **none, Pe** | 11,408 | **0,0001** |
| **Feeding** | **Bacterial Challenge (SexAr)** | **none, Rm** | 8,8216 | **0,0001** |
| **Feeding** | **Bacterial Challenge (SexAr)** | **Va, Pe** | 5,8774 | **0,0001** |
| **Feeding** | **Bacterial Challenge (SexAr)** | **Va, Rm** | 2,4643 | **0,015** |
| **Feeding** | **Bacterial Challenge (SexAr)** | **Pe, Rm** | 4,2423 | **0,0001** |
| **Budding** | **Polyp** | **native polyp, sterile polyp** | 3,6811 | **0,0003** |
| **Budding** | **Bacterial Challenge (SexAr)** | **none, Va** | 8,6968 | **0,0001** |
| **Budding** | **Bacterial Challenge (SexAr)** | **none, Pe** | 9,7257 | **0,0001** |
| **Budding** | **Bacterial Challenge (SexAr)** | **none, Rm** | 9,0516 | **0,0001** |
| **Budding** | **Bacterial Challenge (SexAr)** | **Va, Pe** | 1,438 | 0,1705 |
| **Budding** | **Bacterial Challenge (SexAr)** | **Va, Rm** | 0,41448 | 0,6843 |
| **Budding** | **Bacterial Challenge (SexAr)** | **Pe, Rm** | 1,0838 | 0,2987 |
| **Strobila production** | **Polyp** | **native polyp, sterile polyp** | 10,714 | **0,0001** |
| **Strobila production** | **Bacterial Challenge (SexAr)** | **none, Va** | 12,936 | **0,0001** |
| **Strobila production** | **Bacterial Challenge (SexAr)** | **none, Pe** | 12,767 | **0,0001** |
| **Strobila production** | **Bacterial Challenge (SexAr)** | **none, Rm** | 10,365 | **0,0001** |
| **Strobila production** | **Bacterial Challenge (SexAr)** | **Va, Pe** | 0,95809 | 0,3623 |
| **Strobila production** | **Bacterial Challenge (SexAr)** | **Va, Rm** | 3,5652 | **0,0002** |
| **Strobila production** | **Bacterial Challenge (SexAr)** | **Pe, Rm** | 2,876 | **0,0024** |
| **Ephyrae release** | **Polyp** | **native polyp, sterile polyp** | 13,114 | **0,0001** |
| **Ephyrae release** | **Bacterial Challenge (SexAr)** | **none, Va** | 5,1792 | **0,0001** |
| **Ephyrae release** | **Bacterial Challenge (SexAr)** | **none, Pe** | 5,3148 | **0,0001** |
| **Ephyrae release** | **Bacterial Challenge (SexAr)** | **none, Rm** | 3,4269 | **0,0008** |
| **Ephyrae release** | **Bacterial Challenge (SexAr)** | **Va, Pe** | Denominator is 0 |  |
| **Ephyrae release** | **Bacterial Challenge (SexAr)** | **Va, Rm** | 2,8051 | **0,0025** |
| **Ephyrae release** | **Bacterial Challenge (SexAr)** | **Pe, Rm** | 2,8785 | **0,0005** |

| **Fitness variable** | **Interaction** | **Comparison** | **Factor (Level)** | | | |
| --- | --- | --- | --- | --- | --- | --- |
|  |  |  | **Polyp (native)** | | **Polyp (sterile)** | |
|  |  |  | **t** | **P(perm)** | **t** | **P(perm)** |
| **Survival (Harmed and Dead Pol Count)** | **PoxCh(SexAr)** | **none, Va** | 6,7889 | 0,0001 | 2,0823 | **0,0123** |
| **Survival (Harmed and Dead Pol Count)** | **PoxCh(SexAr)** | **none, Pe** | 8,915 | 0,0001 | 2,7328 | **0,0005** |
| **Survival (Harmed and Dead Pol Count)** | **PoxCh(SexAr)** | **none, Rm** | 6,8863 | 0,0001 | 1,2838 | 0,202 |
| **Survival (Harmed and Dead Pol Count)** | **PoxCh(SexAr)** | **Va, Pe** | 2,0838 | 0,0159 | 0,58761 | 0,6994 |
| **Survival (Harmed and Dead Pol Count)** | **PoxCh(SexAr)** | **Va, Rm** | 1,2765 | 0,2015 | 1,1526 | 0,2895 |
| **Survival (Harmed and Dead Pol Count)** | **PoxCh(SexAr)** | **Pe, Rm** | 2,4964 | 0,0032 | 1,3811 | 0,1473 |
| **Growth** | **PoxCh(SexAr)** | **none, Va** | 0,71171 | 0,4761 | 2,2371 | **0,0265** |
| **Growth** | **PoxCh(SexAr)** | **none, Pe** | 0,35828 | 0,7221 | 0,17555 | 0,8629 |
| **Growth** | **PoxCh(SexAr)** | **none, Rm** | 1,4885 | 0,1404 | 3,5968 | **0,001** |
| **Growth** | **PoxCh(SexAr)** | **Va, Pe** | 0,3771 | 0,7113 | 2,0698 | **0,0413** |
| **Growth** | **PoxCh(SexAr)** | **Va, Rm** | 0,77996 | 0,4383 | 1,3902 | 0,1681 |
| **Growth** | **PoxCh(SexAr)** | **Pe, Rm** | 1,2973 | 0,1912 | 3,3233 | **0,0017** |
| **Strobila production** | **PoxCh(SexAr)** | **none, Va** | 10,717 | 0,0001 | 7,3852 | **0,0001** |
| **Strobila production** | **PoxCh(SexAr)** | **none, Pe** | 11,096 | 0,0001 | 6,5023 | **0,0001** |
| **Strobila production** | **PoxCh(SexAr)** | **none, Rm** | 7,7701 | 0,0001 | 7,2945 | **0,0001** |
| **Strobila production** | **PoxCh(SexAr)** | **Va, Pe** | 0,19132 | 0,8443 | 1,6946 | 0,2451 |
| **Strobila production** | **PoxCh(SexAr)** | **Va, Rm** | 3,4873 | 0,0003 | 0,97386 | 1 |
| **Strobila production** | **PoxCh(SexAr)** | **Pe, Rm** | 3,7209 | 0,0003 | 1,1433 | 0,373 |
| **Ephyrae release** | **PoxCh(SexAr)** | **none, Va** | 4,9711 | 0,0001 | 1,7091 | 0,2444 |
| **Ephyrae release** | **PoxCh(SexAr)** | **none, Pe** | 5,0978 | 0,0001 | 1,755 | 0,2485 |
| **Ephyrae release** | **PoxCh(SexAr)** | **none, Rm** | 3,1204 | 0,0036 | 1,755 | 0,2421 |
| **Ephyrae release** | **PoxCh(SexAr)** | **Va, Pe** | Denominator is 0 |  | Denominator is 0 |  |
| **Ephyrae release** | **PoxCh(SexAr)** | **Va, Rm** | 2,8425 | 0,007 | Denominator is 0 |  |
| **Ephyrae release** | **PoxCh(SexAr)** | **Pe, Rm** | 2,9149 | 0,0036 | Denominator is 0 |  |

| **Fitness variable** | **Factors** | | | | | | **Interaction** | |
| --- | --- | --- | --- | --- | --- | --- | --- | --- |
|  | **Polyp** | | **Seawater** | | **Food (Seawater)** | | **Polyp x Seawater** | |
|  | **Pseudo-F** | **P(perm)** | **Pseudo-F** | **P(perm)** | **Pseudo-F** | **P(perm)** | **Pseudo-F** | **P(perm)** |
| **Survival (Harmed and Dead Pol Count)** | 32,375 | **0,0001** | 7,621 | **0,0005** | 34,533 | **0,0001** | 4,1374 | **0,0147** |
| **Growth** | ns | ns | 5,2151 | **0,0249** | ns | ns | ns | ns |
| **Feeding** | 141,28 | **0,0001** | 24,026 | **0,0001** | 84,087 | **0,0001** | 68,973 | **0,0001** |
| **Budding** | ns | ns | 16,312 | **0,0002** | 33,969 | **0,0001** | 12,562 | **0,0006** |
| **Strobila production** | 77,964 | **0,0001** | 14,215 | **0,0003** | ns | ns | ns | ns |
| **Ephyrae release** | 83,688 | **0,0001** | 9,2068 | **0,0037** | ns | ns | 12,478 | **0,001** |

***D***

| **Fitness variable: Survival (Harmed and Dead Pol Count)** | | | | |
| --- | --- | --- | --- | --- |
| **Comparison** | **t** | | **P(perm)** | |
| native seawater, sterile seawater | 2,7606 | | **0,0008** | |
| native food, sterile food | 5,5966 | | **0,0001** | |
| native polyp, sterile polyp | 5,6899 | | **0,0001** | |
|  | **Polyp x Seawater** | |  | |
|  | **t** | | **P(perm)** | |
|  | **Polyp native** | |  | |
| native seawater, sterile seawater | 1,2684 | | 0,2243 | |
|  | **Polyp sterile** | |  | |
| native seawater, sterile seawater | 1,6286 | | **0,0686** | |
| **Fitness variable: Growth** | | | | |
|  | **Polyp native** | |  | |
| **Comparison** | **t** | | **P(perm)** | |
| native seawater, sterile seawater | 2,4863 | | **0,0128** | |
|  | **Polyp sterile** | |  | |
| native seawater, sterile seawater | ns | | ns | |
| **Fitness variable: Feeding** | | | | |
| **Comparison** | **t** | | **P(perm)** | |
| native seawater, sterile seawater | 4,9016 | | **0,0001** | |
| native food, sterile food | 8,5442 | | **0,0001** | |
| native polyp, sterile polyp | 11,886 | | **0,0001** | |
|  | **Polyp x Seawater** | |  | |
|  | **t** | | **P(perm)** | |
|  | **Polyp native** | |  | |
| native seawater, sterile seawater | 7,1997 | | **0,0001** | |
|  | **Polyp sterile** | |  | |
| native seawater, sterile seawater | 1,934 | | 0,0544 | |
| **Fitness variable: Budding** | | | | |
| **Comparison** | **t** | | **P(perm)** | |
| native seawater, sterile seawater | 4,0388 | | **0,0002** | |
| native food, sterile food | 6,0342 | | **0,0001** | |
|  | **Polyp x Seawater** | |  | |
|  | **t** | | **P(perm)** | |
|  | **Seawater sterile, Polyp sterile** | |  | |
| native food, sterile food | 5,7707 | | **0,0001** | |
|  | **Native food, Seawater native** | |  | |
| native polyp, sterile polyp | 3 | | **0,004** | |
|  | **Sterile food, Seawater sterile** | |  | |
| native polyp, sterile polyp | ns | | ns | |
| **Fitness variable: Strobila** | | | | |
| **Comparison** | **t** | | **P(perm)** | |
| native polyp, sterile polyp | 8,8297 | | **0,0001** | |
| native seawater, sterile seawater | 3,7703 | | **0,0006** | |
| **Fitness variable: Ephyrae** | | | | |
| **Comparison** | **t** | | **P(perm)** | |
| native polyp, sterile polyp | 9,1481 | | **0,0001** | |
| native seawater, sterile seawater | 3,0343 | | **0,0029** | |
| **Polyp x Seawater** | | | | |
|  | **t** | | **P(perm)** | |
| **Polyp native** | | | | |
| native seawater, sterile seawater | 2,977 | | **0,0045** | |
| **Polyp sterile** | | | | |
| native seawater, sterile seawater | ns | | ns | |
|  | |  | |  |

***E***

| **Fitness variable** | Factors | | | | Interaction | |
| --- | --- | --- | --- | --- | --- | --- |
|  | **Polyp** | | **Bacterial Challenge** | | **Polyp X Challenge** | |
|  | **Pseudo-F** | **P(perm)** | **Pseudo-F** | **P(perm)** | **Pseudo-F** | **P(perm)** |
| **Survival (Harmed and Dead Pol Count)** | ns | ns | 15,898 | **0,0001** | 4,1802 | **0,0002** |
| **Growth** | 40,21 | **0,0001** | ns | ns | 3,4681 | **0,017** |
| **Feeding** | 4,7307 | **0,0303** | 50,876 | **0,0001** | ns | ns |
| **Budding** | ns | ns | 71,341 | **0,0001** | 3,1258 | **0,0274** |
| **Strobila production** | 44,061 | **0,0001** | 121,65 | **0,0001** | 19,053 | **0,0001** |
| **Ephyrae release** | 27,061 | **0,0001** | 19,686 | **0,0001** | 13,759 | **0,0001** |

| PAIR-WISE TESTS: Bacterial Challenge data set (2-factorial, fully crossed) | | |
| --- | --- | --- |
| **Fitness variable: Survival (Harmed and Dead Pol Count)** | | |
|  | **Bacterial Challenge** | |
| **Comparison** | **t** | **P(perm)** |
| n, Va | 4,8469 | **0,0001** |
| n, Pe | 7,6262 | **0,0001** |
| n, Rm | 5,1976 | **0,0001** |
| Va, Pe | 1,5862 | 0,0815 |
| Va, Rm | 1,5015 | 0,1031 |
| Pe, Rm | 2,5755 | **0,0007** |
|  | **Polyp native** | |
| n, Va | 6,9215 | **0,0001** |
| n, Pe | 9,1028 | **0,0001** |
| n, Rm | 7,0832 | **0,0001** |
| Va, Pe | 2,1077 | 0,0156 |
| Va, Rm | 1,2353 | 0,2162 |
| Pe, Rm | 2,3385 | **0,0065** |
|  | **Polyp sterile** | |
| n, Va | 1,9921 | **0,017** |
| n, Pe | 2,6915 | **0,0001** |
| n, Rm | 1,2419 | 0,2092 |
| Va, Pe | 0,63783 | 0,6618 |
| Va, Rm | 1,09E+00 | 0,3154 |
| Pe, Rm | 1,3692 | 0,1553 |
| **Fitness variable: Growth** | | |
|  | **Polyp native vs. sterile** | |
| **Factor (Level)** | **t** | **P(perm)** |
| Bac challenge (none) | 1,4954 | 0,1388 |
| Bac challenge (Va) | 3,6023 | **0,0006** |
| Bac challenge (Pe) | 1,9422 | 0,0561 |
| Bac challenge (Rm) | 6,1408 | **0,0001** |
|  | **Polyp native** | |
| **Comparison** | **t** | **P(perm)** |
| n, Va | 0,76367 | 0,4399 |
| n, Pe | 0,40705 | 0,6805 |
| n, Rm | 1,6722 | 0,0965 |
| Va, Pe | 0,3771 | 0,7111 |
| Va, Rm | 0,77996 | 0,4361 |
| Pe, Rm | 1,2973 | 0,1996 |
|  | **Polyp sterile** | |
| **Comparison** | **t** | **P(perm)** |
| n, Va | 2,3208 | **0,0212** |
| n, Pe | 0,17435 | 0,867 |
| n, Rm | 3,9226 | **0,0003** |
| Va, Pe | 2,0698 | **0,0435** |
| Va, Rm | 1,3902 | 0,1691 |
| Pe, Rm | 3,3233 | **0,0012** |
| **Fitness variable: Feeding** | | |
|  | **Bacterial Challenge** | |
| **Comparison** | **t** | **P(perm)** |
| n, Va | 5,4774 | **0,0001** |
| n, Pe | 11,408 | **0,0001** |
| n, Rm | 8,8216 | **0,0001** |
| Va, Pe | 5,8774 | **0,0001** |
| Va, Rm | 2,4643 | **0,0146** |
| Pe, Rm | 4,2423 | **0,0002** |
|  | **Polyp** | |
| native polyp, sterile polyp | 2,175 | **0,0317** |
| **Fitness variable: Budding** | | |
|  | **Bacterial Challenge** | |
| **Comparison** | **t** | **P(perm)** |
| n, Va | 8,6968 | **0,0001** |
| n, Pe | 9,7257 | **0,0001** |
| n, Rm | 9,0516 | **0,0001** |
| Va, Pe | 1,438 | 0,1631 |
| Va, Rm | 0,41448 | 0,6858 |
| Pe, Rm | 1,0838 | 0,2876 |
|  | **Polyp native** | |
| n, Va | 5,7937 | **0,0001** |
| n, Pe | 6,7397 | **0,0001** |
| n, Rm | 6,2787 | **0,0001** |
| Va, Pe | 0,85014 | 0,5843 |
| Va, Rm | 0,50982 | 0,804 |
| Pe, Rm | 0,30593 | 1 |
|  | **Polyp sterile** | |
| n, Va | 6,5458 | **0,0001** |
| n, Pe | 7,1587 | **0,0001** |
| n, Rm | 6,6415 | **0,0001** |
| Va, Pe | 1,3473 | 0,5005 |
| Va, Rm | 2,26E-08 | 1 |
| Pe, Rm | 1,7506 | 0,2422 |
| **Fitness variable: Strobila** | | |
|  | **Bacterial Challenge** | |
| **Comparison** | **t** | **P(perm)** |
| n, Va | 12,936 | **0,0001** |
| n, Pe | 12,767 | **0,0001** |
| n, Rm | 10,365 | **0,0001** |
| Va, Pe | 0,95809 | 0,3588 |
| Va, Rm | 3,5652 | **0,0005** |
| Pe, Rm | 2,876 | **0,0047** |
|  | **Polyp native** | |
| n, Va | 10,717 | **0,0001** |
| n, Pe | 11,096 | **0,0001** |
| n, Rm | 7,7701 | **0,0001** |
| Va, Pe | 0,19132 | 0,8425 |
| Va, Rm | 3,4873 | **0,0007** |
| Pe, Rm | 3,7209 | **0,0006** |
|  | **Polyp sterile** | |
| n, Va | 7,3852 | **0,0001** |
| n, Pe | 6,5023 | **0,0001** |
| n, Rm | 7,2945 | **0,0001** |
| Va, Pe | 1,6946 | 0,2438 |
| Va, Rm | 0,97386 | 1 |
| Pe, Rm | 1,1433 | 0,3658 |
| **Fitness variable: Ephyrae production** | | |
|  | **Bacterial Challenge** | |
| **Comparison** | **t** | **P(perm)** |
| n, Va | 5,1792 | **0,0001** |
| n, Pe | 5,3148 | **0,0001** |
| n, Rm | 3,4269 | **0,0002** |
| Va, Pe | Denominator is 0 |  |
| Va, Rm | 2,8051 | **0,0031** |
| Pe, Rm | 2,8785 | **0,0017** |
|  | **Polyp native** | |
| n, Va | 4,9711 | **0,0001** |
| n, Pe | 5,0978 | **0,0001** |
| n, Rm | 3,1204 | **0,0026** |
| Va, Pe | Denominator is 0 |  |
| Va, Rm | 2,8425 | **0,0071** |
| Pe, Rm | 2,9149 | **0,0028** |
|  | **Polyp sterile** | |
| n, Va | 1,7091 | 0,2471 |
| n, Pe | 1,755 | 0,2414 |
| n, Rm | 1,755 | 0,251 |
| Va, Pe | Denominator is 0 |  |
| Va, Rm | Denominator is 0 |  |
| Pe, Rm | Denominator is 0 |  |
