# Supplementary material for: The Native Microbiome is Crucial for Offspring Generation and Fitness of Aurelia aurita
Source: mBio. 2020 Nov 17;11(6):e02336-20. doi: 10.1128/mBio.02336-20 (PMC7683396; doi:10.1128/mBio.02336-20)
Supplement: TABLE S2 [file mBio.02336-20-st002.docx]

**Tab. S2: Summary of mean fitness status of *A. aurita* polyps and strobilae for all treatments.** Heat map summarizes the significant decreases measured within the treatment groups compared to the native treatment. Based on individual results after host-fitness experiments, monitored fitness parameter survival, growth, budding, feeding, strobilation, and ephyrae release are categorized as (◼) unaffected, (◼) slightly affected, and (◼) crucially affected for each treatment. Additionally, timelines for generation of strobilae and ephyrae release are depicted as days of first appearance after strobilation induction with the synthetic inducer; na, not applicable.

| **Treatment** | **survival** | **growth** | **budding** | | **feeding** | **strobilation** | | | | **ephyrae release** |  |  |
| --- | --- | --- | --- | --- | --- | --- | --- | --- | --- | --- | --- | --- |
|  |  |  |  |  |  | **early strobila** | | | **late strobila** |  |  |  |
| **native** |  |  |  | |  | 5 d | | | 9 d | 12 d |  |  |
| **native in sterile environment** |  |  |  | |  | 8 d | | | 17 d | 22 d |  |  |
| **sterile in native environment** |  |  |  | |  | 11 d | | | na | na |  |  |
| **sterile with native food** |  |  |  | |  | 10 d | | | na | na |  |  |
| **sterile** |  |  |  | |  | 7 d | | | 14 d | na |  |  |
| **native + *V. anguillarum*** |  |  |  | |  | na | | | na | na |  |  |
| **sterile + *V. anguillarum*** |  |  |  | |  | na | | | na | na |  |  |
| **native + *P. espejiana*** |  |  |  | |  | na | | | na | na |  |  |
| **sterile + *P. espejiana*** |  |  |  | |  | na | | | na | na |  |  |
| **native + *R. mobilis*** |  |  |  | |  | 14 d | | | 21 d | 27 d |  |  |
| **sterile + *R. mobilis*** |  |  |  | |  | na | | | na | na |  |  |
| **re-colonized** |  |  |  | |  | 5 d | | | 10 d | 14 d |  |  |
|  |  | | |  | | |  |  | | | |  |
