# Supplementary material for: The Native Microbiome is Crucial for Offspring Generation and Fitness of Aurelia aurita
Source: mBio. 2020 Nov 17;11(6):e02336-20. doi: 10.1128/mBio.02336-20 (PMC7683396; doi:10.1128/mBio.02336-20)
Supplement: FIG S2 [file mBio.02336-20-sf002.docx]

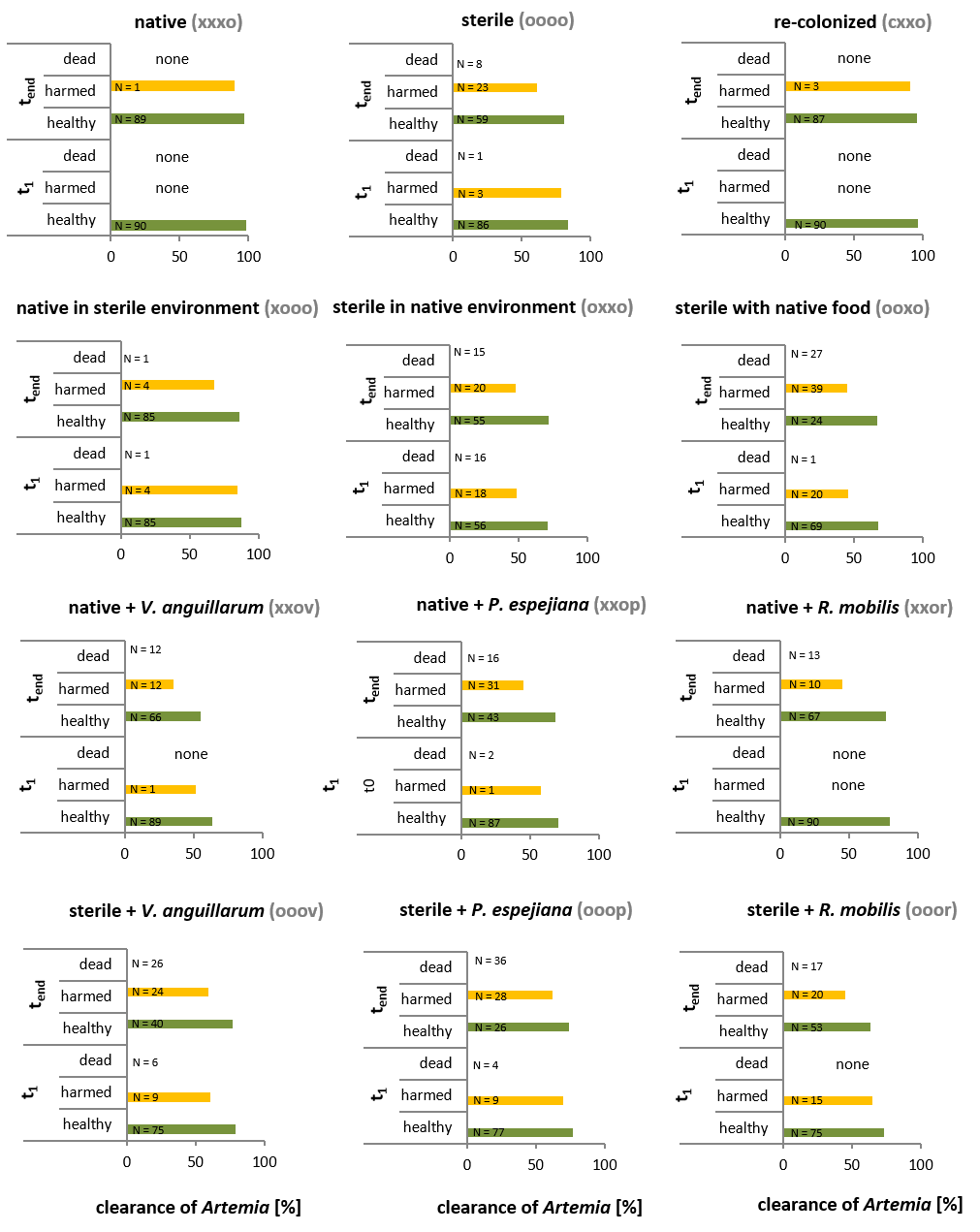


**Fig. S2: Clearance of *Artemia salina* by *A. aurita* polyps.** Feeding rate as clearance of *A. salina* in percentages. Single polyps were incubated for 1 h with 20 *A. salina*. After incubation, remaining *A. salina* were counted to calculate the feeding rate of healthy as well as harmed polyps for all treatments (corresponding code in brackets). Compared are the results of day 1 (t_1_) and day 5 (t_end_).
