# Supplementary material for: The Native Microbiome is Crucial for Offspring Generation and Fitness of Aurelia aurita
Source: mBio. 2020 Nov 17;11(6):e02336-20. doi: 10.1128/mBio.02336-20 (PMC7683396; doi:10.1128/mBio.02336-20)
Supplement: FIG S3 [file mBio.02336-20-sf003.docx]

***A***


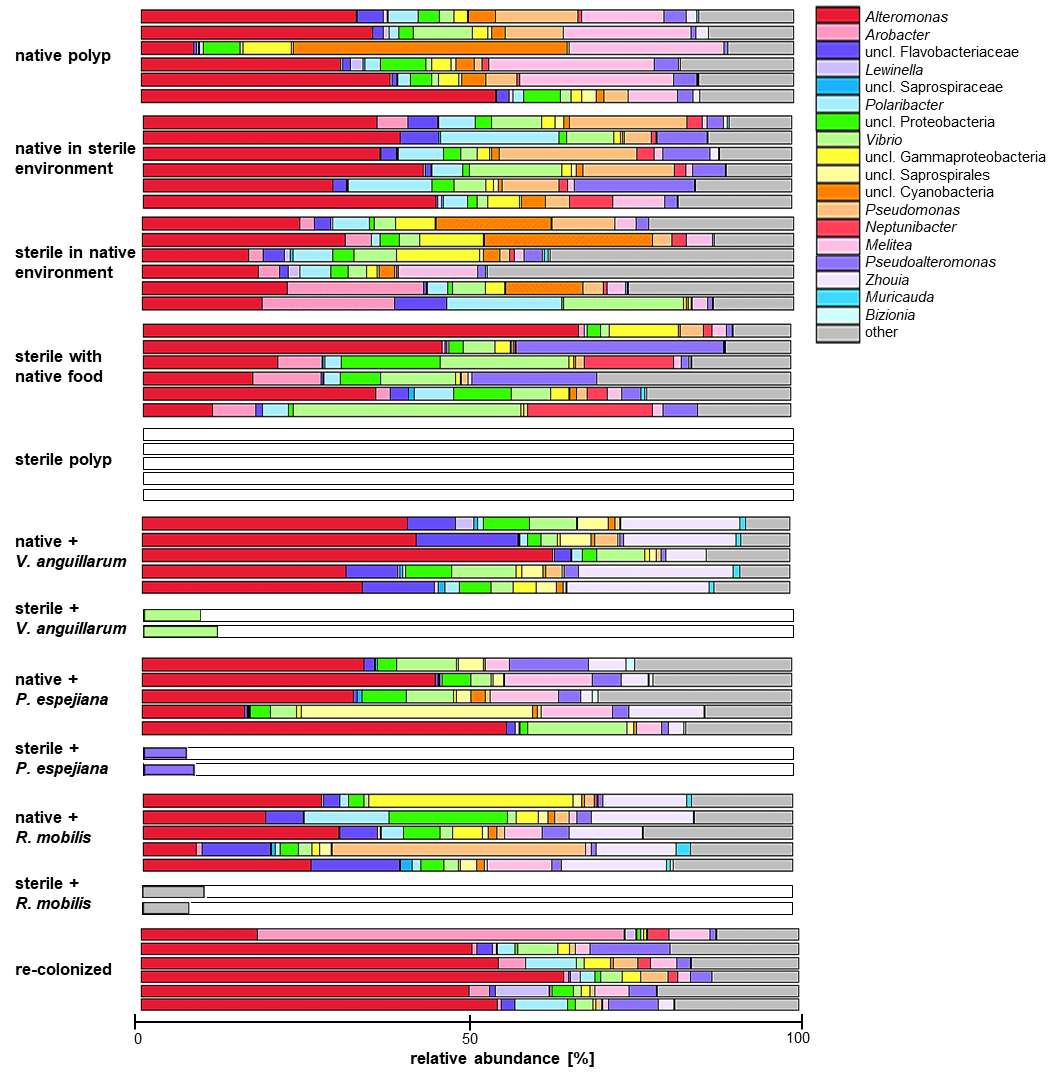


***B***


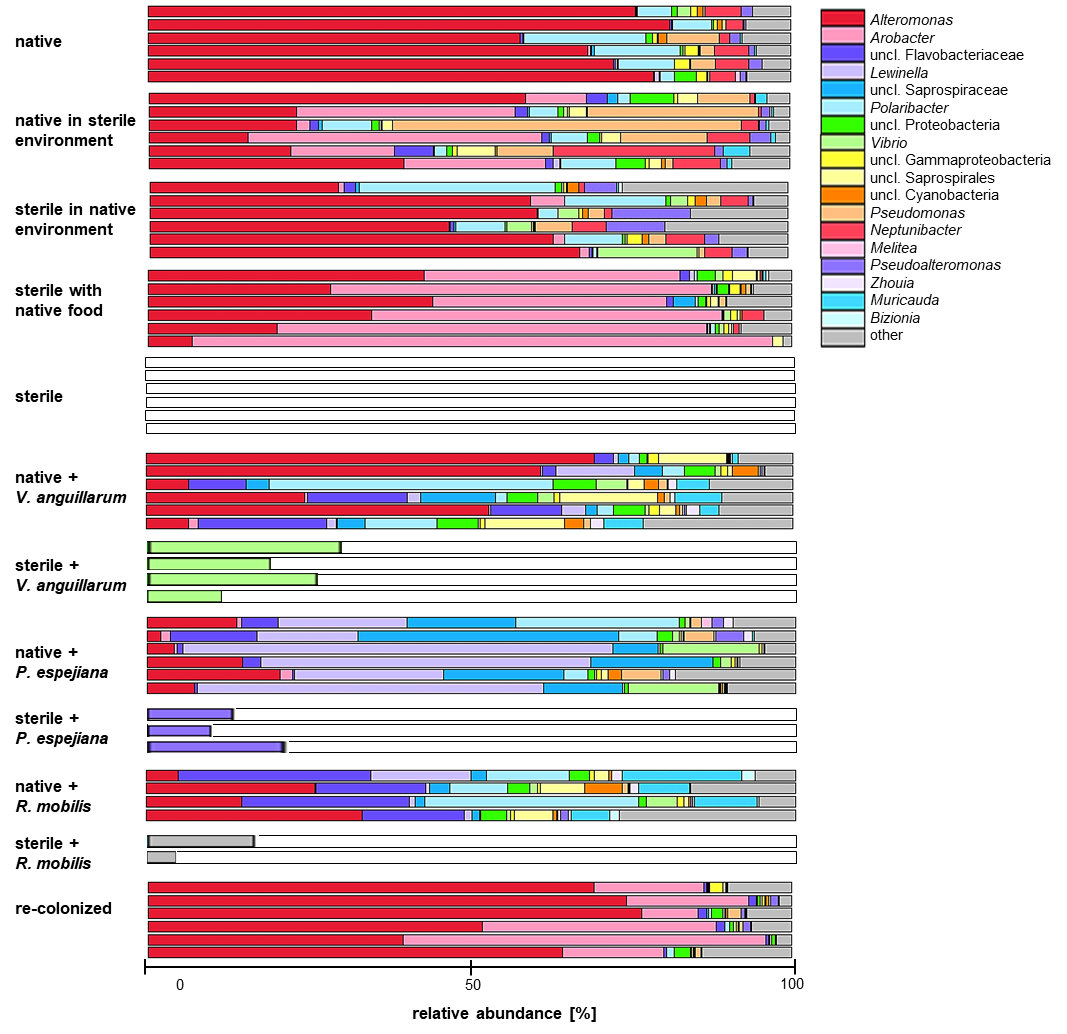


**Fig. S3: Microbial community composition of *A. aurita* in the experimental treatment groups.** Microbial communities were analyzed by sequencing the V1-V2 region of 16S bacterial rRNA genes. OTU abundances were summarized at the genus level and normalized by the total number of reads per sample. Bar plots are grouped according to sample type, each group including 2 - 6 replicates. Microbial community patterns of (***A***) polyps and (***B***) strobilae.

(***A***)
