# Supplementary material for: The Native Microbiome is Crucial for Offspring Generation and Fitness of Aurelia aurita
Source: mBio. 2020 Nov 17;11(6):e02336-20. doi: 10.1128/mBio.02336-20 (PMC7683396; doi:10.1128/mBio.02336-20)
Supplement: FIG S4 [file mBio.02336-20-sf004.docx]

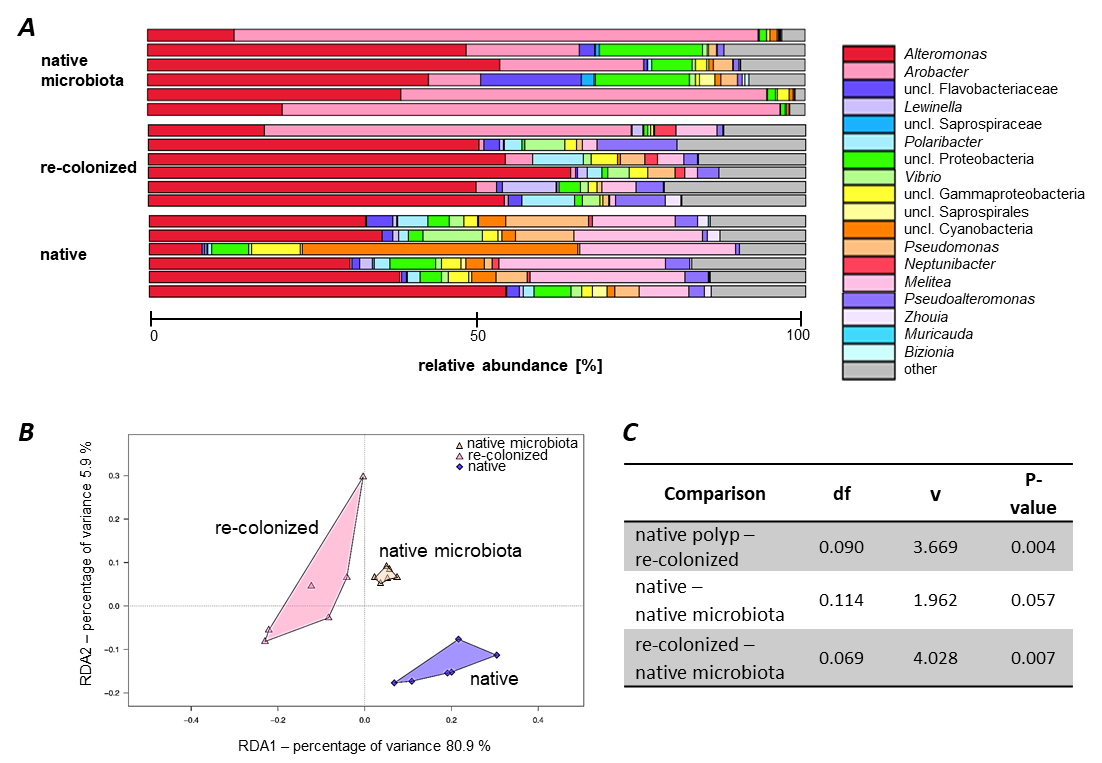


**Fig. S4: Microbial composition of re-colonized polyps.** Composition of microbiota associated with native and re-colonized *A. aurita* polyps as well as of the generated native microbiota for re-colonization of sterile polyps. Microbial communities were analyzed by sequencing the V1-V2 region of 16S bacterial rRNA genes. (***A***) OTU abundances were summarized at the genus level and normalized by the total number of reads per sample. Bar plots are grouped according to sample type, each group including 6 replicates. (***B***) Redundancy analysis plots of Hellinger-transformed OTU abundances. Each point represents the whole microbial community of the respective sample, replicates of one sample group are framed by polygons. (***C***) Results of pairwise tests in beta diversity analysis. Tests were conducted for specified comparisons. df, degree of freedom; v, variance.
