# Supplementary material for: The Native Microbiome is Crucial for Offspring Generation and Fitness of Aurelia aurita
Source: mBio. 2020 Nov 17;11(6):e02336-20. doi: 10.1128/mBio.02336-20 (PMC7683396; doi:10.1128/mBio.02336-20)
Supplement: TABLE S3 [file mBio.02336-20-st003.docx]

**Tab. S3: Results of pairwise tests in beta diversity analysis.** Permutation test for RDA under reduced model was conducted with permutations (Number of permutations: 9999) for all comparisons, but only specified comparisons are shown. Model: RDA (formula = OTUhel ~ Group, data = metaDataSub); df = 0.321, v = 6.663, p-value =0.00001.

| Comparison | df | v | p-value |
| --- | --- | --- | --- |
| polyps | | | |
| native – native in sterile environment | 0.066 | 3.373 | 0.010 |
| native – sterile in native environment | 0799 | 2.986 | 0.005 |
| native – sterile with native food | 0.124 | 5.008 | 0.005 |
| native – re-colonized | 0.153 | 2.130 | 0.014 |
| native – native + *V. anguillarum* | 0.132 | 6.947 | 0.003 |
| native – sterile + *V. anguillarum* | 0.146 | 4.053 | 0.045 |
| native – native + *P. espejiana* | 0.101 | 5.445 | 0.001 |
| native – sterile + *P. espejiana* | 0.086 | 2.742 | 0.039 |
| native – native + *R. mobilis* | 0.103 | 4.034 | 0.003 |
| native – sterile + *R. mobilis* | 0.173 | 3.997 | 0.032 |
| native in sterile environment – sterile in native environment | 0.101 | 3.831 | 0.005 |
| sterile in native environment – sterile with native food | 0.119 | 3.776 | 0.002 |
| native + *V. anguillarum* – sterile + *V. anguillarum* | 0.174 | 7.170 | 0.054 |
| native + *P. espejiana* – sterile + *P. espejiana* | 0.156 | 4.118 | 0.036 |
| native + *R. mobilis* – sterile + *R. mobilis* | 0.173 | 3.335 | 0.048 |
| native + *V. anguillarum* – native + *P. espejiana* | 0.108 | 2.971 | 0.008 |
| native + *V. anguillarum* – native + *R. mobilis* | 0.054 | 2.168 | 0.010 |
| native + *P. espejiana* – native + *R. mobilis* | 0.102 | 2.993 | 0.008 |
| strobilae | | | |
| native – native in sterile environment | 0.143 | 9.046 | 0.001 |
| native – sterile in native environment | 0.027 | 2.259 | 0.003 |
| native – sterile with native food | 0.223 | 15.757 | 0.004 |
| native – re-colonized | 0.144 | 3.856 | 0.002 |
| native – native + *V. anguillarum* | 0.174 | 8.993 | 0.009 |
| native – sterile + *V. anguillarum* | 0.165 | 13.234 | 0.028 |
| native – native + *P. espejiana* | 0.137 | 12.115 | 0.003 |
| native – sterile + *P. espejiana* | 0.168 | 12.742 | 0.051 |
| native – native + *R. mobilis* | 0.133 | 9.034 | 0.004 |
| native – sterile + *R. mobilis* | 0.182 | 9.997 | 0.003 |
| native in sterile environment – sterile in native environment | 0.127 | 6.397 | 0.004 |
| sterile in native environment – sterile with native food | 0.120 | 4.505 | 0.003 |
| native + *V. anguillarum* – sterile + *V. anguillarum* | 0.192 | 7.390 | 0.049 |
| native + *P. espejiana* – sterile + *P. espejiana* | 0.163 | 5.812 | 0.033 |
| native + *R. mobilis* – sterile + *R. mobilis* | 0.137 | 5.397 | 0.009 |
| native + *V. anguillarum* – native + *P. espejiana* | 0.149 | 4.995 | 0.016 |
| native + *V. anguillarum* – native + *R. mobilis* | 0.134 | 4.044 | 0.029 |
| native + *P. espejiana* – native + *R. mobilis* | 0.121 | 4.799 | 0.056 |
