# Supplementary material for: The Native Microbiome is Crucial for Offspring Generation and Fitness of Aurelia aurita
Source: mBio. 2020 Nov 17;11(6):e02336-20. doi: 10.1128/mBio.02336-20 (PMC7683396; doi:10.1128/mBio.02336-20)
Supplement: TABLE S4 [file mBio.02336-20-st004.docx]

**Tab. S4: Correlation of Operational taxonomic units (OTUs) with the fitness status of *A. aurita* polyps.** OTUs listed with their taxonomic classification are correlated with the fitness status (◼) unaffected/healthy, (◼) slightly affected/ marginally harmed, and (◼) crucially affected/ essentially harmed of *A. aurita* polyps. At least 50 % of the biological replicates analyzed were associated with the respective OTU.

| **OTU** | **read counts** | **taxonomic classification** | |
| --- | --- | --- | --- |
|  |  | **phylum** | **genus** |
| OTU0056 | 972 | Actinobacteria | *Propionibacterium* |
| OTU0005 | 815 | Bacteroidetes | *Lewinella* |
| OTU0043 | 119 | Bacteroidetes | *Muricauda* |
| OTU0055 | 991 | Bacteroidetes | *Olleya* |
| OTU0007 | 8549 | Bacteroidetes | *Polaribacter* |
| OTU0079 | 331 | Bacteroidetes | *Polaribacter* |
| OTU0024 | 542 | Bacteroidetes | *Roseivirga* |
| OTU0027 | 2476 | Bacteroidetes | uncl. Flavobacteriaceae |
| OTU0088 | 680 | Bacteroidetes | uncl. Flavobacteriales |
| OTU0008 | 3677 | Bacteroidetes | uncl. Saprospirales |
| OTU0061 | 864 | Bacteroidetes | uncl. Bacteroidetes |
| OTU0040 | 791 | Bacteroidetes | uncl. BME43 |
| OTU0032 | 98 | Bacteroidetes | uncl. Flavobacteriaceae |
| OTU0049 | 1581 | Bacteroidetes | uncl. Flavobacteriales |
| OTU0131 | 157 | Bacteroidetes | uncl. Flavobacteriales |
| OTU0006 | 479 | Bacteroidetes | uncl. Saprospiraceae |
| OTU0017 | 10442 | Bacteroidetes | *Zhouia* |
| OTU0009 | 7130 | Cyanobacteria | uncl. Cyanobacteria |
| OTU0035 | 1081 | Firmicutes | *Staphylococcus* |
| OTU0037 | 2251 | Proteobacteria | *Alcanivorax* |
| OTU0001 | 1843 | Proteobacteria | *Alteromonas* |
| OTU0002 | 11694 | Proteobacteria | *Alteromonas* |
| OTU0003 | 8634 | Proteobacteria | *Alteromonas* |
| OTU0033 | 2436 | Proteobacteria | *Alteromonas* |
| OTU0096 | 7 | Proteobacteria | *Alteromonas* |
| OTU0014 | 9815 | Proteobacteria | *Arcobacter* |
| OTU0083 | 620 | Proteobacteria | *Glaciecola* |
| OTU0016 | 4439 | Proteobacteria | *Melitea* |
| OTU0120 | 384 | Proteobacteria | *Melitea* |
| OTU0070 | 958 | Proteobacteria | *Methylotenera* |
| OTU0015 | 3294 | Proteobacteria | *Neptuniibacter* |
| OTU0031 | 2180 | Proteobacteria | *Oleibacter* |
| OTU0134 | 40 | Proteobacteria | *Pseudoalteromonas* |
| OTU0019 | 9601 | Proteobacteria | *Pseudoalteromonas* |
| OTU0060 | 433 | Proteobacteria | *Pseudoalteromonas* |
| OTU0010 | 11100 | Proteobacteria | *Pseudomonas* |
| OTU0067 | 440 | Proteobacteria | uncl. Francisellaceae |
| OTU0018 | 4266 | Proteobacteria | uncl. Gammaproteobacteria |
| OTU0038 | 785 | Proteobacteria | uncl. Gammaproteobacteria |
| OTU0044 | 805 | Proteobacteria | uncl. Gammaproteobacteria |
| OTU0074 | 1521 | Proteobacteria | uncl. J115 |
| OTU0020 | 5347 | Proteobacteria | uncl. Proteobacteria |
| OTU0042 | 161 | Proteobacteria | uncl. Proteobacteria |
| OTU0062 | 188 | Proteobacteria | uncl. Proteobacteria |
| OTU0149 | 164 | Proteobacteria | uncl. Proteobacteria |
| OTU0220 | 87 | Proteobacteria | uncl. Sinobacteraceae |
| OTU0013 | 9280 | Proteobacteria | *Vibrio* |
| OTU0028 | 1920 | Proteobacteria | *Vibrio* |
| OTU0039 | 321 | Proteobacteria | *Vibrio* |
| OTU0078 | 1353 | Proteobacteria | *Vibrio* |
| OTU0047 | 1654 | TM6 | uncl. SJA-4 |
| OTU0034 | 1162 | Bacteroidetes | *Crocinitomix* |
| OTU0112 | 20 | Bacteroidetes | *Crocinitomix* |
| OTU0046 | 254 | Bacteroidetes | *Maribacter* |
| OTU0030 | 772 | Bacteroidetes | uncl. Cytophagales |
| OTU0011 | 2562 | Bacteroidetes | uncl. Flavobacteriaceae |
| OTU0004 | 1004 | Proteobacteria | *Arcobacter* |
| OTU0130 | 69 | Proteobacteria | *Bacteriovorax* |
| OTU0281 | 28 | Proteobacteria | *Glaciecola* |
| OTU0135 | 46 | Proteobacteria | *Porticoccus* |
| OTU0263 | 26 | Proteobacteria | *Spongiibacter* |
| OTU0041 | 36 | Proteobacteria | uncl. Bacteriovoracaceae |
| OTU0179 | 53 | Proteobacteria | *Ruegeria* |
| OTU0267 | 43 | Proteobacteria | uncl. Gammaproteobacteria |
| OTU0121 | 66 | Proteobacteria | uncl. Oceanospirillaceae |
| OTU0026 | 311 | Proteobacteria | uncl. Proteobacteria |
| OTU0051 | 31 | Proteobacteria | uncl. Proteobacteria |
| OTU0377 | 13 | Actinobacteria | *Corynebacterium* |
| OTU0163 | 15 | Actinobacteria | *Microbacterium* |
| OTU0115 | 27 | Actinobacteria | *Microbacterium* |
| OTU0166 | 28 | Actinobacteria | *Micrococcus* |
| OTU0175 | 29 | Actinobacteria | *Rothia* |
| OTU0200 | 35 | Bacteria | uncl. Bacteria |
| OTU0036 | 110 | Bacteroidetes | *Arenibacter* |
| OTU0141 | 14 | Bacteroidetes | *Balneola* |
| OTU0021 | 138 | Bacteroidetes | *Bizionia* |
| OTU0218 | 14 | Bacteroidetes | *Fluviicola* |
| OTU0025 | 166 | Bacteroidetes | *Kriegella* |
| OTU0054 | 4 | Bacteroidetes | *Lewinella* |
| OTU0023 | 292 | Bacteroidetes | *Muricauda* |
| OTU0241 | 24 | Bacteroidetes | *Polaribacter* |
| OTU0128 | 65 | Bacteroidetes | *Polaribacter* |
| OTU0154 | 22 | Bacteroidetes | *Porphyromonas* |
| OTU0142 | 39 | Bacteroidetes | *Prevotella* |
| OTU0225 | 64 | Bacteroidetes | *Roseivirga* |
| OTU0045 | 308 | Bacteroidetes | *Ulvibacter* |
| OTU0022 | 719 | Bacteroidetes | uncl. Cryomorphaceae |
| OTU0102 | 231 | Bacteroidetes | uncl. Cyclobacteriaceae |
| OTU0093 | 39 | Bacteroidetes | uncl. Cytophagales |
| OTU0082 | 20 | Bacteroidetes | uncl. Flavobacteriaceae |
| OTU0155 | 17 | Bacteroidetes | uncl. Flavobacteriaceae |
| OTU0097 | 45 | Bacteroidetes | uncl. Saprospiraceae |
| OTU0145 | 39 | Firmicutes | *Lactococcus* |
| OTU0338 | 19 | Firmicutes | *Peptoniphilus* |
| OTU0162 | 22 | Firmicutes | *Staphylococcus* |
| OTU0161 | 26 | Firmicutes | *Streptococcus* |
| OTU0103 | 30 | Firmicutes | *Streptococcus* |
| OTU0169 | 60 | Firmicutes | uncl. Mogibacteriaceae |
| OTU0065 | 911 | Proteobacteria | *Arcobacter* |
| OTU0107 | 128 | Proteobacteria | *Chromobacterium* |
| OTU0137 | 205 | Proteobacteria | *Legionella* |
| OTU0236 | 18 | Proteobacteria | *Melitea* |
| OTU0077 | 36 | Proteobacteria | *Nannocystis* |
| OTU0099 | 76 | Proteobacteria | *Oleibacter* |
| OTU0116 | 76 | Proteobacteria | *Oleibacter* |
| OTU0048 | 26 | Proteobacteria | *Plesiocystis* |
| OTU0098 | 72 | Proteobacteria | *Pseudoalteromonas* |
| OTU0248 | 73 | Proteobacteria | *Ruegeria* |
| OTU0138 | 49 | Proteobacteria | uncl. Gammaproteobacteria |
| OTU0052 | 25 | Proteobacteria | uncl. Proteobacteria |
| OTU0258 | 24 | Proteobacteria | uncl. Proteobacteria |
| OTU0355 | 21 | Proteobacteria | uncl. Proteobacteria |
| OTU0203 | 86 | Proteobacteria | *Vibrio* |
