# Supplementary material for: The Native Microbiome is Crucial for Offspring Generation and Fitness of Aurelia aurita
Source: mBio. 2020 Nov 17;11(6):e02336-20. doi: 10.1128/mBio.02336-20 (PMC7683396; doi:10.1128/mBio.02336-20)
Supplement: TABLE S5 [file mBio.02336-20-st005.docx]

**Tab. S5: Correlation of Operational taxonomic units (OTUs) with the segmentation of *A. aurita* polyps and ephyrae release.** (◼) Unaffected, (◼) slightly affected, and (◼) crucially affected formation of strobilae and further ephyrae release are correlated with respective listed OTUs. At least 50 % of the biological replicates analyzed were associated with the respective OTU.

| **OTU** | **read counts** | **taxonomic classification** | |
| --- | --- | --- | --- |
|  |  | **phylum** | **genus** |
| OTU0106 | 63 | Actinobacteria | *Corynebacterium* |
| OTU0377 | 62 | Actinobacteria | *Corynebacterium* |
| OTU0115 | 22 | Actinobacteria | *Microbacterium* |
| OTU0056 | 266 | Actinobacteria | *Propionibacterium* |
| OTU0090 | 370 | Bacteria | uncl. Bacteria |
| OTU0036 | 36 | Bacteroidetes | *Arenibacter* |
| OTU0021 | 253 | Bacteroidetes | *Bizionia* |
| OTU0040 | 14 | Bacteroidetes | uncl. BME43 |
| OTU0034 | 166 | Bacteroidetes | *Crocinitomix* |
| OTU0233 | 168 | Bacteroidetes | *Crocinitomix* |
| OTU0178 | 15 | Bacteroidetes | uncl. JTB248 |
| OTU0025 | 23 | Bacteroidetes | *Kriegella* |
| OTU0005 | 21 | Bacteroidetes | *Lewinella* |
| OTU0046 | 715 | Bacteroidetes | *Maribacter* |
| OTU0071 | 62 | Bacteroidetes | *Maribacter* |
| OTU0023 | 47 | Bacteroidetes | *Muricauda* |
| OTU0043 | 53 | Bacteroidetes | *Muricauda* |
| OTU0007 | 34 | Bacteroidetes | *Polaribacter* |
| OTU0079 | 9 | Bacteroidetes | *Polaribacter* |
| OTU0154 | 415 | Bacteroidetes | *Porphyromonas* |
| OTU0024 | 20 | Bacteroidetes | *Roseivirga* |
| OTU0058 | 37 | Bacteroidetes | *Sediminicola* |
| OTU0045 | 25 | Bacteroidetes | *Ulvibaacter* |
| OTU0061 | 32 | Bacteroidetes | uncl. Bacteroidetes |
| OTU0022 | 9 | Bacteroidetes | uncl. Cryomorphaceae |
| OTU0102 | 59 | Bacteroidetes | uncl. Cyclobacteriaceae |
| OTU0030 | 19 | Bacteroidetes | uncl. Cytophagales |
| OTU0011 | 138 | Bacteroidetes | uncl. Flavobacteriaceae |
| OTU0012 | 56 | Bacteroidetes | uncl. Flavobacteriaceae |
| OTU0027 | 2534 | Bacteroidetes | uncl. Flavobacteriaceae |
| OTU0032 | 437 | Bacteroidetes | uncl. Flavobacteriaceae |
| OTU0049 | 113 | Bacteroidetes | uncl. Flavobacteriales |
| OTU0088 | 19 | Bacteroidetes | uncl. Flavobacteriales |
| OTU0006 | 408 | Bacteroidetes | uncl. Saprospiraceae |
| OTU0008 | 43 | Bacteroidetes | uncl. Saprospirales |
| OTU0017 | 18 | Bacteroidetes | *Zhouia* |
| OTU0183 | 57 | Cyanobacteria | *Chloroidium* |
| OTU0009 | 130 | Cyanobacteria | uncl. Cyanobacteria |
| OTU0114 | 221 | Firmicutes | *Anaerococcus* |
| OTU0080 | 384 | Firmicutes | *Finegoldia* |
| OTU0336 | 11 | Firmicutes | *Peptoniphilus* |
| OTU0035 | 128 | Firmicutes | *Staphylococcus* |
| OTU0169 | 145 | Firmicutes | uncl. Mogibacteriaceae |
| OTU0037 | 14 | Proteobacteria | *Alcanivorax* |
| OTU0001 | 63 | Proteobacteria | *Alteromonas* |
| OTU0002 | 62 | Proteobacteria | *Alteromonas* |
| OTU0003 | 22 | Proteobacteria | *Alteromonas* |
| OTU0033 | 266 | Proteobacteria | *Alteromonas* |
| OTU0004 | 370 | Proteobacteria | *Arcobacter* |
| OTU0014 | 36 | Proteobacteria | *Arcobacter* |
| OTU0177 | 253 | Proteobacteria | *Enhydrobacter* |
| OTU0229 | 14 | Proteobacteria | *Haemophilus* |
| OTU0137 | 166 | Proteobacteria | *Legionella* |
| OTU0057 | 168 | Proteobacteria | *Marinobacter* |
| OTU0016 | 15 | Proteobacteria | *Melitea* |
| OTU0120 | 23 | Proteobacteria | *Melitea* |
| OTU0070 | 21 | Proteobacteria | *Methylotenera* |
| OTU0015 | 715 | Proteobacteria | *Neptuniibacter* |
| OTU0031 | 62 | Proteobacteria | *Oleibacter* |
| OTU0019 | 47 | Proteobacteria | *Pseudoalteromonas* |
| OTU0060 | 53 | Proteobacteria | *Pseudoalteromonas* |
| OTU0010 | 34 | Proteobacteria | *Pseudomonas* |
| OTU0125 | 9 | Proteobacteria | *Ruegeria* |
| OTU0029 | 415 | Proteobacteria | *Shewanella* |
| OTU0069 | 20 | Proteobacteria | *Spongiibacter* |
| OTU0041 | 37 | Proteobacteria | uncl. Bacteriovoracaceae |
| OTU0081 | 25 | Proteobacteria | uncl. Comamonadaceae |
| OTU0066 | 32 | Proteobacteria | uncl. Enterobacteriaceae |
| OTU0018 | 9 | Proteobacteria | uncl. Gammaproteobacteria |
| OTU0038 | 59 | Proteobacteria | uncl. Gammaproteobacteria |
| OTU0044 | 19 | Proteobacteria | uncl. Gammaproteobacteria |
| OTU0095 | 138 | Proteobacteria | uncl. J115 |
| OTU0020 | 56 | Proteobacteria | uncl. Proteobacteria |
| OTU0026 | 2534 | Proteobacteria | uncl. Proteobacteria |
| OTU0042 | 437 | Proteobacteria | uncl. Proteobacteria |
| OTU0050 | 113 | Proteobacteria | uncl. Proteobacteria |
| OTU0062 | 19 | Proteobacteria | uncl. Proteobacteria |
| OTU0086 | 408 | Proteobacteria | uncl. Proteobacteria |
| OTU0013 | 43 | Proteobacteria | *Vibrio* |
| OTU0028 | 18 | Proteobacteria | *Vibrio* |
| OTU0039 | 57 | Proteobacteria | *Vibrio* |
| OTU0047 | 130 | TM6 | uncl. SJA-4 |
| OTU0141 | 221 | Bacteroidetes | *Balneola* |
| OTU0268 | 384 | Bacteroidetes | *Lewinella* |
| OTU0085 | 11 | Bacteroidetes | SC3-56 |
| OTU0132 | 128 | Bacteroidetes | uncl. Cryomorphaceae |
| OTU0059 | 145 | Bacteroidetes | uncl. Flammeovirgaceae |
| OTU0162 | 14 | Firmicutes | *Staphylococcus* |
| OTU0129 | 63 | Proteobacteria | *Alcanivorax* |
| OTU0094 | 62 | Proteobacteria | *Alteromonas* |
| OTU0083 | 22 | Proteobacteria | *Glaciecola* |
| OTU0192 | 266 | Proteobacteria | HTCC |
| OTU0260 | 370 | Proteobacteria | *Neptuniibacter* |
| OTU0099 | 36 | Proteobacteria | *Oleibacter* |
| OTU0116 | 253 | Proteobacteria | *Oleibacter* |
| OTU0108 | 14 | Proteobacteria | uncl. Gammaproteobacteria |
| OTU0167 | 166 | Proteobacteria | uncl. Gammaproteobacteria |
| OTU0092 | 168 | Proteobacteria | uncl. Oceanospirillales |
| OTU0051 | 15 | Proteobacteria | uncl. Proteobacteria |
| OTU0052 | 23 | Proteobacteria | uncl. Proteobacteria |
| OTU0226 | 21 | Actinobacteria | *Corynebacterium* |
| OTU0163 | 715 | Actinobacteria | *Microbacterium* |
| OTU0175 | 62 | Actinobacteria | *Rothia* |
| OTU0075 | 47 | Bacteria | uncl. Bacteria |
| OTU0170 | 53 | Bacteria | uncl. Bacteria |
| OTU0117 | 34 | Bacteroidetes | *Balneola* |
| OTU0105 | 9 | Bacteroidetes | *Bizionia* |
| OTU0242 | 415 | Bacteroidetes | *Bizionia* |
| OTU0218 | 20 | Bacteroidetes | *Fluviicola* |
| OTU0054 | 37 | Bacteroidetes | *Lewinella* |
| OTU0084 | 25 | Bacteroidetes | *Lewinella* |
| OTU0101 | 32 | Bacteroidetes | *Lewinella* |
| OTU0158 | 9 | Bacteroidetes | *Maribacter* |
| OTU0055 | 59 | Bacteroidetes | *Olleya* |
| OTU0142 | 19 | Bacteroidetes | *Prevotella* |
| OTU0064 | 138 | Bacteroidetes | uncl. Bacteroidetes |
| OTU0270 | 56 | Bacteroidetes | uncl. Bacteroidetes |
| OTU0082 | 2534 | Bacteroidetes | uncl. Flavobacteriaceae |
| OTU0118 | 437 | Bacteroidetes | uncl. Flavobacteriaceae |
| OTU0152 | 113 | Bacteroidetes | uncl. Flavobacteriaceae |
| OTU0182 | 19 | Bacteroidetes | uncl. Flavobacteriaceae |
| OTU0223 | 408 | Bacteroidetes | uncl. Flavobacteriaceae |
| OTU0072 | 43 | Bacteroidetes | uncl. Flavobacteriales |
| OTU0089 | 18 | Bacteroidetes | uncl. Flavobacteriales |
| OTU0068 | 57 | Bacteroidetes | uncl. Saprospiraceae |
| OTU0300 | 130 | Firmicutes | *Gemella* |
| OTU0250 | 221 | Firmicutes | *Granulicatella* |
| OTU0374 | 384 | Firmicutes | *Peptoniphilus* |
| OTU0103 | 11 | Firmicutes | *Streptococcus* |
| OTU0161 | 128 | Firmicutes | *Streptococcus* |
| OTU0255 | 145 | Firmicutes | *Streptococcus* |
| OTU0286 | 14 | Firmicutes | *Streptococcus* |
| OTU0202 | 63 | Firmicutes | *Veillonella* |
| OTU0432 | 62 | Fusobacteria | *Fusobacterium* |
| OTU0144 | 22 | Proteobacteria | *Alteromonas* |
| OTU0252 | 266 | Proteobacteria | *Alteromonas* |
| OTU0164 | 370 | Proteobacteria | *Arcobacter* |
| OTU0324 | 36 | Proteobacteria | *Arcobacter* |
| OTU0063 | 253 | Proteobacteria | *Bacteriovorax* |
| OTU0077 | 14 | Proteobacteria | *Nannocystis* |
| OTU0098 | 166 | Proteobacteria | *Pseudoalteromonas* |
| OTU0283 | 168 | Proteobacteria | uncl. Deltaproteobacteria |
| OTU0067 | 15 | Proteobacteria | uncl. Francisellaceae |
| OTU0053 | 23 | Proteobacteria | uncl. Gammaproteobacteria |
| OTU0110 | 21 | Proteobacteria | uncl. Gammaproteobacteria |
| OTU0138 | 715 | Proteobacteria | uncl. Gammaproteobacteria |
| OTU0179 | 62 | Proteobacteria | uncl. *Rmgeria* |
| OTU0173 | 47 | Proteobacteria | uncl. Haliangiaceae |
| OTU0074 | 53 | Proteobacteria | uncl. J115 |
| OTU0343 | 34 | Proteobacteria | uncl. Oceanospirillaceae |
| OTU0100 | 9 | Proteobacteria | uncl. Proteobacteria |
| OTU0203 | 415 | Proteobacteria | *Vibrio* |
| OTU0216 | 20 | Verrucomicrobia | *Cerasicoccus* |
