# Supplementary material for: The Native Microbiome is Crucial for Offspring Generation and Fitness of Aurelia aurita
Source: mBio. 2020 Nov 17;11(6):e02336-20. doi: 10.1128/mBio.02336-20 (PMC7683396; doi:10.1128/mBio.02336-20)
Supplement: FIG S5 [file mBio.02336-20-sf005.docx]

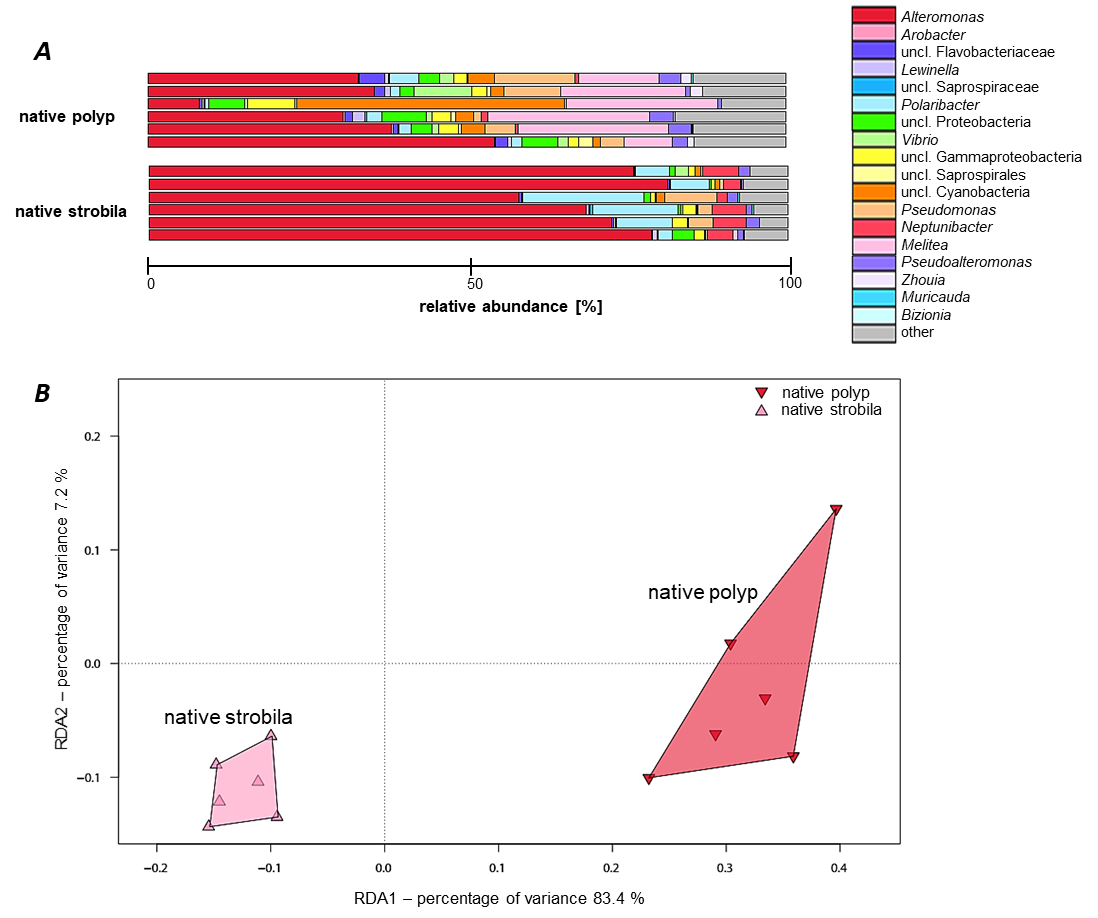


**Fig. S5: Microbial composition of strobilae.** Composition of microbiota associated with native *A. aurita* polyps before strobilation induction and native late strobilae 9 days after induction of strobilation with 5 µM 5-methoxy-2-methyl indole. Microbial communities were analyzed by sequencing the V1-V2 region of 16S bacterial rRNA genes. (***A***) OTU abundances were summarized at the genus level. (***B***) Redundancy analysis plots of Hellinger-transformed OTU abundances (df = 0.136, v = 9.652, p-value = 0.005).
